# Quorum Sensing Modulates the Epibiotic-Parasitic Relationship Between *Actinomyces odontolyticus* and Its Saccharibacteria epibiont, a *Nanosynbacter lyticus* Strain, TM7x

**DOI:** 10.3389/fmicb.2018.02049

**Published:** 2018-09-24

**Authors:** Joseph K. Bedree, Batbileg Bor, Lujia Cen, Anna Edlund, Renate Lux, Jeffrey S. McLean, Wenyuan Shi, Xuesong He

**Affiliations:** ^1^Section of Oral Biology, Division of Oral Biology and Medicine, School of Dentistry, University of California, Los Angeles, Los Angeles, CA, United States; ^2^Department of Microbiology, The Forsyth Institute, Cambridge, MA, United States; ^3^Department of Genomic Medicine, J. Craig Venter Institute, La Jolla, CA, United States; ^4^Section of Periodontics, Division of Constitutive and Regenerative Sciences, School of Dentistry, University of California, Los Angeles, Los Angeles, CA, United States; ^5^Department of Periodontics, School of Dentistry, University of Washington, Seattle, WA, United States

**Keywords:** *Actinomyces*, TM7, human-associated, epibiont, oral microbiome, interspecies interaction

## Abstract

The ultra-small, obligate parasitic epibiont, TM7x, the first and only current member of the long-elusive Saccharibacteria (formerly the TM7 phylum) phylum to be cultivated, was isolated in co-culture with its bacterial host, *Actinomyces odontolyticus* subspecies *actinosynbacter*, XH001. Initial phenotypic characterization of the TM7x-associated XH001 co-culture revealed enhanced biofilm formation in the presence of TM7x compared to XH001 as monoculture. Genomic analysis and previously published transcriptomic profiling of XH001 also revealed the presence of a putative AI-2 quorum sensing (QS) operon, which was highly upregulated upon association of TM7x with XH001. This analysis revealed that the most highly induced gene in XH001 was an *lsrB* ortholog, which encodes a putative periplasmic binding protein for the auto inducer (AI)-2 QS signaling molecule. Further genomic analyses suggested the *lsrB* operon in XH001 is a putative hybrid AI-2/ribose transport operon as well as the existence of a *luxS* ortholog, which encodes the AI-2 synthase. In this study, the potential role of AI-2 QS in the epibiotic-parasitic relationship between XH001 and TM7x in the context of biofilm formation was investigated. A genetic system for XH001 was developed to generate *lsrB* and *luxS* gene deletion mutants in XH001. Phenotypic characterization demonstrated that deletion mutations in either *lsrB* or *luxS* did not affect XH001’s growth dynamic, mono-species biofilm formation capability, nor its ability to associate with TM7x. TM7x association with XH001 induced *lsrB* gene expression in a *luxS-*dependent manner. Intriguingly, unlike wild type XH001, which displayed significantly increased biofilm formation upon establishing the epibiotic-parasitic relationship with TM7x, XH001Δ*lsrB*, and XH001Δ*luxS* mutants failed to achieve enhanced biofilm formation when associated with TM7x. In conclusion, we demonstrated a significant role for AI-2 QS in modulating dual-species biofilm formation when XH001 and TM7x establish their epibiotic-parasitic relationship.

## Introduction

One of the greatest scientific revelations in recent history was the discovery of the enormous diversity and large abundance of microbes associated with the human body. These microbial communities co-evolve with humans and have important roles in health and disease. However, only a limited proportion of these microbial species can be cultured and studied *in vitro*, an observation known as the “Great Plate Anomaly,” (Staley and Konopka, 1985; Rappé and Giovannoni, 2003). Recently, a unique and intimate association between two oral bacterial isolates from different phyla, a *Nanosynbacter lyticus* Type Strain TM7x HMT-952 (TM7x), a member of the Saccharibacteria/TM7 phylum that has an ultra-small cell size (McLean et al., 2018), and an *Actinomyces odontolyticus* subspecies *actinosynbacter* strain “XH001," was discovered (He et al., 2015; McLean et al., 2016). Co-isolation of these strains revealed TM7x to be an obligate epibiotic parasite, which lives on the surface of its bacterial host, XH001. This interbacterial interaction represents a novel relationship in the bacterial kingdom yet to be characterized on a molecular level. Importantly, TM7x is the only cultivated member to date of the recently discovered Candidate Phyla Radiation (CPR) (Hug et al., 2016) organisms that could account for upward of 26% of domain bacteria (Brown et al., 2015; Castelle and Banfield, 2018). Overlapping characteristics between the phyla comprising of the CPR organisms include reduced genomes, lack of the biosynthetic capacity for most amino acids, as well as ultra-small cell morphological sizes inferred from representatives of the WWE3, OP11, and OD1 phyla, which are capable of passing through 0.2-μm-sized filters (Luef et al., 2015). This suggests that a proportion of other CPR representatives may exist in epibiotic-parasitic relationships comparable to TM7x with XH001. Therefore, it is imperative to characterize this interaction on a molecular level to uncover important knowledge for studying other “yet-to-be cultivated" CPR bacterial species.

As early colonizers, *Actinomyces* species contribute to oral microbial biofilm formation along with *Streptococci* (Nyvad and Kilian, 1987, 1990; Diaz et al., 2006; Dige et al., 2007, 2009a,b; Ding et al., 2010) and are associated with several human diseases including actinomycosis, periodontitis, oral carcinoma, and childhood caries (Nagy et al., 1998; Smego and Foglia, 1998; Becker et al., 2002; Aas et al., 2005; Colombo et al., 2006; Kanasi et al., 2010; Ling et al., 2010; Sato et al., 2012). Similarly, Saccharibacteria members are implicated in several human inflammatory-mucosal diseases, such as inflammatory bowel disease, periodontitis, and vaginosis (Paster et al., 2001; Fredricks et al., 2005; Kuehbacher et al., 2008; Kianoush et al., 2014; Soro et al., 2014). Saccharibacteria phylum members are particularly prevalent in the oral community in a low abundance of ∼1% in the health-associated oral microbial community, with an increase up to 21% in patients with various types of periodontitis (Paster et al., 2002; Brinig et al., 2003; Kumar et al., 2003; Rylev et al., 2011; Liu et al., 2012). Furthermore, certain oral Saccharibacteria phylotypes are detected on or within mammalian host crevicular epithelial cells, a hallmark indicator of bacterial pathogenesis in periodontal disease (Paster et al., 2002). Our initial characterization allowed the first glimpse into the epibiotic-parasitic nature of TM7x when associated with XH001, which induces reciprocal morphological changes in XH001 and TM7x as well as differential gene expression patterns in XH001 (He et al., 2015; Bor et al., 2016). Interestingly, association with TM7x repressed the XH001-induced TNF-α mRNA expression in macrophages, implying that TM7x may either mask surface protein expression in XH001 required for induction of TNF-α by macrophages or directly suppress TNF-α expression in macrophages (He et al., 2015). This intriguing relationship provides an ideal model for unraveling the underpinnings of the epibiotic-parasitic relationship, and its ecological function in the oral microbiome, as well as the role in oral health and disease.

Our recent transcriptomic profiling (He et al., 2015) revealed that during association of XH001 with TM7x, the most highly induced XH001 gene is an *lsrB* ortholog, which encodes a putative periplasmic binding protein for the auto inducer (AI)-2 quorum sensing (QS) signaling molecule. Despite numerous species identified to produce the AI-2 signal (Kolenbrander et al., 2010), only three types of AI-2 receptors have been characterized in detail. These include LuxP (*Vibrio harveyi*), LsrB (*Salmonella enterica* serovar Typhimurium str. 14028, *Escherichia coli* MG1655, *Sinorhizobium meliloti*, and *Aggregatibacter actinomycetecommitans* HK1651), and RbsB (*A. actinomycetecommitans* HK1651) (Bassler et al., 1994; Taga et al., 2001, 2003; Xavier and Bassler, 2005; Shao et al., 2007a,b; Pereira et al., 2008). AI-2 production has been reported in the literature to be regulated in a positive feedback loop: as AI-2 is transported into the cell in a cell density-depended manner, *luxS* expression is upregulated, leading to increased production of AI-2 to achieve quorum followed by rapid internalization during stationary phase to activate the QS operon and downstream regulated genes (Taga et al., 2001, 2003; Wang et al., 2005). In addition to mediating multispecies biofilm formation (Bassler et al., 1997) in the oral cavity (Rickard et al., 2006; Periasamy and Kolenbrander, 2009; Kolenbrander et al., 2010), AI-2 QS has also been widely shown to regulate genes controlling virulence factor production, particularly in commensal and pathogenic bacteria, including oral representatives such as *Porphyromonas gingivalis* (Chung et al., 2001; Burgess et al., 2002), *A. actinomycetecommitans* (Fong et al., 2001, 2003), *Streptococcus intermedius* (Pecharki et al., 2008; Ahmed et al., 2009), *Streptococcus mutans* (Merritt et al., 2003, 2005), and *Streptococcus gordonii* (McNab et al., 2003). In this study, the role of AI-2 QS in the epibiotic-parasitic relationship between TM7x and XH001 was investigated.

## Materials and Methods

### Bacterial Strains, Plasmids, and Media

All bacterial strains and plasmids used in this study and their characteristics are listed in **Table 1**. TM7x associated *A. odontolyticus* subspecies *actinosynbacter* strain XH001, XH001Δ*lsrB*, and XH001Δ*luxS* and as monospecies were incubated in Brain-Heart Infusion (BHI) broth or agar (Difco Laboratories, Detroit, Michigan) at 37°C in microaerophilic conditions (2% O_2_, 5% CO_2_, balanced with Nitrogen) using a Whitely Workstation A35 (Microbiology International). All monoculture XH001, XH001Δ*lsrB*, and XH001Δ*luxS* were infected with TM7x using an established protocol (He et al., 2015). Media were supplemented with 150 μg/mL kanamycin sulfate (Fischer Bioreagents, Hampton, NH, United States) for antibiotic selection when appropriate. Positive and negative *V. harveyi* control strains, BB152 and MM77, respectively, used in the GC-MS analysis of AI-2 were grown in AI Bioassay medium (AB) for ∼14 h shaking (215 rpm) at 30°C as described in an established protocol (Greenberg et al., 1979; Taga and Xavier, 2005).

**Table 1:** Bacterial strains and plasmids used in this study.

Strain/Plasmid	Relevant Characteristics	Reference
pJRD215	Broad-host range expression vector, Km^R^ Sm^R^ Mob^+^, 10.2 kb	Davison et al., 1987
XH001	WT, *A. odontolyticus* subspecies *actinosynbacter* strain, Km^S^	He et al., 2015
XH001+TM7x	WT, TM7x-associated *A. odontolyticus* subspecies *actinosynbacter* strain, Km^S^	He et al., 2015
XH001Δ*lsrB*	Δ*lsrB* Km^R^ in XH001 background	This Study
XH001Δ*lsrB*+TM7x	TM7x-associated XH001Δ*lsrB*	This Study
XH001Δ*luxS*	Δ*luxS* Km^R^ in XH001 background	This Study
XH001Δ*luxS*+TM7x	TM7x-associated XH001Δ*luxS*	This Study
BB152	*luxLM*::Tn5 in *Vibrio harveyi* strain	Bassler et al., 1994
MM77	*luxLM*::Tn*5*, *luxS*::Cm^R^ in *Vibrio harveyi* strain	Mok et al., 2003

### Genetic and Molecular Phylogenetic Analyses of the LsrB and LuxS Orthologs in XH001

The closed annotated XH001 genome (accession no. LLVT00000000-the version described in this paper is version LLVT00000000.1.) was previously published (McLean et al., 2016) and provided the predicted protein sequences used in the NCBI-Blastp, NCBI Conserved Domain Database (Marchler-Bauer et al., 2010, 2014, 2016), and PHYRE2 (Kelley and Sternberg, 2009) analyses. Protein sequence alignment and phylogenetic analyses (construct via maximum-likelihood method) were conducted using the ClustalW Omega alignment method through MEGA7 software (Jones et al., 1992; Sievers et al., 2011; Kumar et al., 2016). The LsrB, RbsB, and LuxS protein accession numbers are listed in **Supplementary Table 2**.

### PCR, Cloning, and Single Chromosomal Deletion Construct Development

The shuttle vector, pJRD215 (Davison et al., 1987; Yeung and Kozelsky, 1994) was isolated using QIAprep Spin Miniprep kit (Qiagen Cat. No./ID 27104). All primers were designed (Integrated DNA Technologies, Inc., Coralville, IA, United States) based upon the sequence data from the published XH001 genome via Primer3 software (Koressaar and Remm, 2007; Untergasser et al., 2012) and are listed in **Supplementary Table 1**. The chromosomal gene deletion constructs were generated using a modified protocol of fusion PCR as described (Shevchuk et al., 2004).

The respective 0.8 kb up/downstream fragments of the *lsrB* and *luxS* genes, as well as the kanamycin gene resistance cassette of pJDR215, were PCR-amplified according to the schematic depicted in **Supplementary Figure 2** using the corresponding primers listed in **Supplementary Table 1**. These fragments were combined in a subsequent fusion reaction in which the overlapping ends annealed. The resulting product was subjected to a final PCR amplification using the manufacturer’s protocol for the Phusion Hi-Fidelity PCR Master Mix with GC Buffer (New England Biolabs) DNA polymerase generating a 2.8-kb construct.

After generating the linear gene deletion construct fragments, they were isolated via gel extraction using the QIAquick Gel Extraction kit (Qiagen, Hilden Germany) following the manufacturer’s instructions and purified using DNA Clean Concentrator Kit (Zymo Research DNA Clean Concentrator Cat. No./ID D4013). Using the manufacturer’s protocol for the Phusion Hi-Fidelity PCR Master Mix with GC Buffer, PCR amplification of the resulting full *lsrB* and *luxS* gene deletion constructs were amplified using primers 1 and 6 and 7 and 12, respectively (**Supplementary Figure 2**), was used to generate sufficient product prior to transformation.

### RNA Isolation and cDNA Synthesis

RNA isolation and cDNA synthesis protocols were performed as previously reported (Bor et al., 2016) with the following modifications: cells were harvested from a 50 mL culture in BHI under 2% microaerophilic conditions after 18 h of incubation. Cells were collected by centrifugation at 4600 rpm for 10 min. Prior to RNA isolation, cell pellets were treated for RNA stabilization with RNA protect Cell Reagent (Qiagen, Cat. No./ID 76526). Total RNA was isolated using RNeasy Protect Bacteria Mini Kit (Qiagen, Cat. No./ID: 74524) following the manufacturer’s protocol. RNA Clean Concentrator Kit (Zymo Research RNA Clean Concentrator Cat. No./ID R1015) was applied for terminal purification of the RNA.

### Quantitative Real-Time PCR Analysis

Quantitative real-time PCR (qRT-PCR) analysis was performed using an established protocol (Bor et al., 2016) with the following modifications: the GeneCopoeia All-in-One qPCR mix (GeneCopoeia, Inc., Rockville, MD, United States) was used and the final qRT-PCR mixture (20 μL) contained 10XGeneCopoeia All in One mix, 1 μg cDNA, and 10 μM of the appropriate forward and reverse qRT-PCR primers designed for the *lsrB* gene (**Supplementary Table 1**). To calculate the relative *lsrB* expression, we used a previously developed protocol (Bor et al., 2016). qRT-PCR analysis of *lsrB* expression was evaluated using *lsrB* primers specific to XH001 (XH001 *lsrB* F and XH001 *lsrB* R) and the relative fold expression of *lsrB* was calculated in reference to the expression of the 16S gene in XH001 using primers specific to the XH001 16S rRNA gene (F5, R3) (**Supplementary Table 1**) described in a previous study (Bor et al., 2016). The sequence of the *lsrB* gene was verifiably absent in the TM7x genome as confirmed via PCR and sequencing (data not shown). Each assay was performed with at least two independent RNA samples in triplicate. One-way ANOVA analysis was used to compare all monoculture and co-culture groups separately for differences in expression rates of *lsrB*. The *t*-test statistical analysis was applied for pairwise comparisons between expression rates of *lsrB* between XH001 and TM7x-associated XH001, TM7x-associated XH001 and TM7x-associated XH001Δ*lsrB*, TM7x-associated XH001 and TM7x-associated XH001Δ*luxS*.

### Electrocompetent Cell Preparation, Transformation Procedure, and Mutant Verification

Fresh competent cells were prepared using a previously described protocol (Yeung and Kozelsky, 1994) with the following modifications: Inoculation of 30 μL of XH001 stock into 2 mL of reduced BHI (2% O_2_). After overnight incubation, cell cultures were diluted 1:10 into 20 mL of fresh BHI. After an additional overnight incubation, cultures were inoculated into 100 mL of fresh BHI to a starting optical density of 0.1 OD_600_. After incubation for 5–8 h early exponential phase cells were harvested (between 0.2 and 0.3 OD_600_). The 100 mL culture was split into 50 mL cultures, chilled for 1.5 h, and centrifuged for 10 min at 4600 rpm in a 4°C refrigerated Sorvall Legend RT centrifuge [Sorvall (United Kingdom) Ltd., Thermo Fischer Scientific]. Harvested cells were washed twice with ice cold _dd_H_2_O and once with 10% glycerol. The washed XH001 cells were resuspended in 10% glycerol (5 × 10^8^ cells/mL) and were transformed with pJRD215 as described (Yeung and Kozelsky, 1994) to demonstrate transformability and expression of the kanamycin cassette in the XH001 background. To generate *lsrB* and *luxS* gene deletion mutants, 1 μg of *lsrB* and *luxS* gene deletion constructs (DNA) was separately added to different XH001 electrocompetent culture suspensions. Transformation was facilitated using chilled 0.2-mm Fischerbrand electroporation cuvettes (Fischer Scientific, Hampton, NH, United States, Cat. No./ID FB102). The following electroporation settings were used: the electroporation was carried out using a Bio-Rad Gene Pulser II with the capacitance set to 25 μF, 2.5 kV, and resistance set to 400 ohms in parallel. The pulsed cells were diluted with reduced, 37°C BHI medium, and recovered for 3 h by incubation in 1 mL of BHI at 37°C in microaerophilic conditions (2% O_2_, 5% CO_2_, balanced with Nitrogen) using a Whitely Workstation A35 (Microbiology International). After recovery, the 1.1 mL culture cells were centrifuged for 10 min at 4600 RPM and resuspended in 200 μL of fresh, reduced, 37°C BHI media, and plated. XH001 transformants were selected using 150 μg/mL kanamycin sulfate on BHI agar plates for up to 6 days. Unpulsed electrocompetent cells or electrocompetent cells without transformable DNA were used as controls. No transformants were obtained without electric pulse or absence of transformable DNA.

XH001Δ*lsrB* and XH001Δ*luxS* mutants were confirmed via DNA sequencing (Laragen, Inc, Culver City, CA, United States): briefly, in the putative XH001Δ*lsrB* and XH001Δ*luxS* mutant backgrounds and wild type XH001, PCR amplification of gDNA, upstream and downstream of the transcriptional start site in the target genes (approximately 500 bp amplicon), determined the presence or absence of the kanamycin resistance cassette in the correct locus. Primers 13/14 and 17/18 were used to amplify a ∼500-bp amplicon from XH001Δ*lsrB* and XH001Δ*luxS* gDNA, respectively using the manufacturer’s protocol for the Phusion Hi-Fidelity PCR Master Mix with GC Buffer. Primers 15/16 and 19/20 were used to amplify a ∼450- to 500-bp amplicon of overlapping sequences of the *lsrB* and *luxS* genes, respectively, from XH001 gDNA. XH001Δ*lsrB* and XH001Δ*luxS* were tested for lack of full mRNA transcript production to further verify true chromosomal deletion. Thus, primers 21/22 and 23/24 were designed to amplify the entire *lsrB* and *luxS* genes, respectively. Transcripts were absent in the XH001Δ*lsrB* and XH001Δ*luxS* background, but present in XH001. An annealing temperature of 58°C was used for all reactions and the PCR cycling parameters were identical except for the annealing temperature used to evaluate XH001Δ*luxS* transcript production, which was set to 57°C for primers 19/20.

### Growth Kinetics and Generation Time

XH001, XH001Δ*lsrB*, and XH001Δ*luxS* as monospecies and associated with TM7x were incubated at 37°C in microaerophilic conditions using a Whitely Workstation A35 (Microbiology International) for growth kinetics analyses using the BioSense Solutions (Farum, Demark) oCelloScope platform (CVR/VAT: 38602926). Wild type XH001, XH001Δ*lsrB*, and XH001Δ*luxS* were started at 0.05 OD_600_ in a final volume of 250 μL in a Corning clear 96-well plate (Corning, Corning, NY, United States). Three independent cultures were each grown in triplicate in two separate experiments. Time course measurements for Background Corrected Absorbance (BCA units), occurred every 30 min for 24 h. The BCA unit output was analyzed by growth kinetic algorithms based upon the measurement of light absorption, which is determined to be more robust and sensitive when compared to traditional optical density methods (Canali et al., 2018). The BCA value was calculated as BCA value = log 10 [∑ (object pixel intensities)]. Generation times and maximum optical density were calculated from these data. One-way ANOVA analysis was used to compare all monoculture groups separately for differences in generation time.

### Phase Contrast Imaging and Confocal Laser Scanning Microscopy (CLSM) Imaging of Biofilm Formation

After the established infection assay (He et al., 2015; Bor et al., 2016) was conducted to generate TM7x-associated XH001, XH001Δ*lsrB* and XH001Δ*luxS* co-cultures, representative phase contrast images were acquired using previously reported parameters (Bor et al., 2016) to capture the nascent TM7x infection of XH001, XH001Δ*lsrB* and XH001Δ*luxS* for evaluation of TM7x infectivity. TM7x-associated XH001, XH001Δ*lsrB* and XH001Δ*luxS* and as monospecies, were cultured associated with TM7x and without in sterile, μ-slide 4 well plastic bottom slide (Ibidi, Martinsried, Germany) for 24 h with a starting OD_600_ of 0.25 prior to analysis. After removal of the supernatant and planktonic cells, the biofilms were stained with 600 μL of sterile SYTO 9 (1:1000 diluted in PBS) solution and visualized with scanning confocal laser microscopy (Zeiss LSM 880, Oberkochen, Germany) using an Argon laser. The Zeiss parameters for image acquisition included a *Z*-slice thickness of 0.350–0.700 μM and a plan-apochromat 63X (N.A = 1.4) objective under oil immersion. *Z*-stacks were imaged with a 488-nm laser line, 488-nm beam splitter, and a wavelength detection range of 503–547 nm. Images were reconstructed using Bitplane: Imaris-Microscopy Image Analysis Software (Bitplane, an Oxford instruments company, Belfast, United Kingdom). Reconstructed images resulted in horizontal (xy) and sagittal (xz) views of the imaged biofilms.

### Quantification of Biofilm Maximal Thickness (Height), Biovolume, Biofilm Roughness Correlation (Variance), and Biofilm Continuity Ratio

Imaris (Bitplane, Belfast, United Kingdom) biofilm analysis XTension software was used to obtain maximal biovolume thickness (height), biovolume, biofilm roughness correlation (variance), and the biofilm continuity ratio. Biovolume thickness measures from the top of the surface to the base (substratum) and includes all gaps present in the reconstructed surface. Biovolume was obtained by measuring the volume of all surface objects (cubic micrometers). The compactness of the biofilm was assessed as total fluorescence per volume of biofilm (Ghafoor et al., 2011). Roughness coefficient (variance) was the measure of the variability of the localized thicknesses relative to the total mean thickness (defining the structured smoothness of the biofilm). The output data resulted from the roughness correlation formula: roughness coefficient (varience) = $\sqrt{\frac{\mathrm{sum}\;[(\mathrm{local thickness}-\mathrm{overall thickness})2]}{\mathrm{Number of local thickness measurements}}}$. Biofilm roughness coefficient = 0, suggested a perfect uniformly thick surface. The larger this variance, the more uneven the surface object was considered. The larger the variance, the rougher a biofilm was considered. Biofilm continuity ratio was a dimensionless coefficient that measured the continuity of the reconstructed surface. It was measured by taking mean surface mask thickness divided by the mean biovolume thickness. A value of “1” represented perfect continuity throughout the *Z*-stack. Values less than one were used to define how many gaps (holes) there were in the surface reconstruction. Surface mask thickness quantifies only those voxels in vertical column that lie inside the reconstructed 3D volume. If there were gaps in the rendering, they were not included in the thickness measure. One-way ANOVA analysis was used to compare all monoculture and co-culture groups separately applied for evaluating differences in maximal biovolume thickness (height), biovolume, biofilm roughness correlation (variance), and biofilm continuity ratio. The *t*-test statistical analysis was applied for pairwise comparisons of maximal biovolume thickness (height), biovolume, biofilm roughness correlation (variance), and biofilm continuity ratio between XH001 and TM7x-associated XH001, TM7x-associated XH001 and TM7x-associated XH001Δ*lsrB*, TM7x-associated XH001, and TM7x-associated XH001Δ*luxS*. Non-associated XH001, XH001Δ*lsrB*, and XH001Δ*luxS* were evaluated and compared via one-way ANOVA to ensure no intrinsic differences were attributed to the *lsrB* or *luxS* mutations.

### Gas Chromatography-Mass Spectrometry Analysis of (S)-4,5-Dihydroxy-2,3-pentanedione (DPD), AI-2

TM7x-associated XH001, XH001Δ*lsrB*, and XH001Δ*luxS* or the respective monocultures were grown as indicated above for 18 h (starting at 0.1 OD_600_) using 5 mL cultures. To prepare cultures for the (S)-4,5-Dihydroxy-2,3-pentandione (DPD, AI-2) analysis, a previous published protocol was used with the following modifications (Thiel et al., 2009): cultures were centrifuged at 4600 × *g* for 10 min and the supernatant was harvested and filter-sterilized with Millipore Millex 0.22-μM sterile syringe filters. Aliquots of 1 mL of supernatant were added to 500 μL of 100 mM Potassium Phosphate buffer (pH 7.2) in glass test tubes. Samples were exposed to solid, 2 mg of 1,2 phenylenediamine (in excess) incubated at room temperature for 2 h (vortexing every 20 min) derivatizing DPD to quinoxalinediol. After derivatization, quinoxalinediol-d4 (deuterated-quinoxalinediol) serving as the internal standard, was added to a final concentration of 250 ng/mL using a Hamilton 5 μL Model 75 glass syringe-32 gauge, 2 inch, point style 3 (Hamilton, Reno, NV, United States). Samples were manually homogenized using sterile, disposable, Fischer brand borosilicate glass (Fischer Scientific, Hampton, NH, United States) Pasteur pipets (Cat. No 22-183632). About 1 mL of dichloromethane (DCM) was added and homogenized. Samples were centrifuged at 4600 × *g* for 5 min to fully separate DCM and H_2_O layer. The DCM layer was harvested through manual liquid/liquid extraction. Samples were then dried under soft flow of nitrogen gas for 1 h at room temperature. 80 μL of N-Methyl-N-(trimethylsilyl)trifuoroacetamide (Cat. No. M7891 CAS No. 24589-78-4) was added to dried samples and incubated in a 65°C water bath for 30 min. After this silylation and terminal derivatization, the samples were analyzed for S-DPD concentration. In addition, the positive and negative controls, BB152 and MM77, respectively, were prepared similarly. Each sample was vortex GC-MS measurements were carried out using an Agilent Model 7683 Autosampler (Agilent Technologies, Inc., Santa Clara, CA, United States), 6890 Gas Chromatograph, and 5975 Inert Mass Selective Detector in the Electron Impact (EI) mode. Sample injection was carried out in splitless mode with a 2-min purge, and inlet temperature set to 280°C. Separation was carried out on an Agilent HP5-MS column with dimensions 30m × 250 μm × 0.25 μm. Ultra High Purity Grade He (Airgas, Radnor, PA, United States) was used as carrier gas with the flow set to 0.9 mL/min in constant flow mode. The initial oven temperature was set to 100°C for 3 min followed by a 25°C/min ramp to a final temperature of 300°C which was maintained for 4 min. A 3.2 min solvent delay was used. EI energy was set to 70 eV. The MSD Enhanced Chemstation software (Agilent) was set to scan the 40–600 m/z range. Data collection and analysis were performed using GC/MSD Chemstation Software (Agilent). S-DPD abundance was determined by measuring the area under the extracted ion traces for m/z 245 and 348 for the derivatized S-DPD, and m/z 249 and 352 for the deuterated standard. Measured S-DPD abundance values were normalized to those of the deuterated internal standard in each sample and OD_600_. Absolute concentration values were determined using an external standard calibration curve in the range 25–250 ng/mL. No carryover was detected using MSTFA-TMCS blanks. Interpolation of DPD, AI-2 concentration was confirmed via reproducibility in derivatization of DPD external standards used in absolute quantification (**Supplementary Figures 4**, **5**). The *t*-test statistical analysis was applied for pairwise comparisons of differences in DPD, AI-2 production between XH001 and TM7x-associated XH001, TM7x-associated XH001 and TM7x-associated XH001Δ*lsrB*, TM7x-associated XH001 and TM7x-associated XH001Δ*luxS*.

## Results

### XH001 Encodes a *lsrB* and a *luxS* Ortholog

**FIGURE 1:**
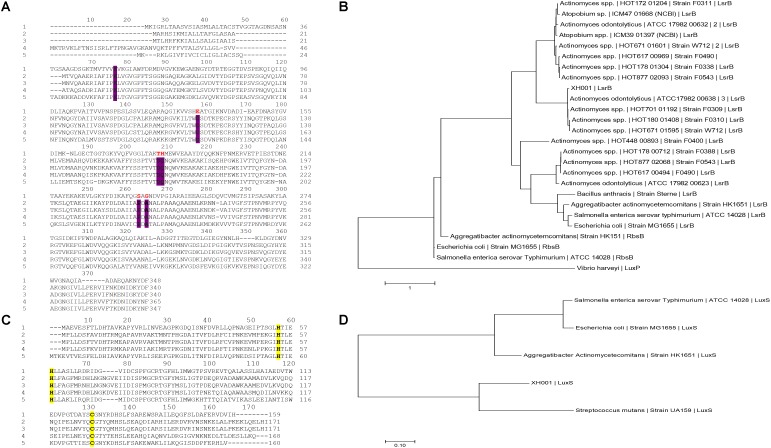
Protein sequence alignment and molecular phylogenetic analyses of the LsrB and LuxS in XH001. **(A)** Protein sequence alignment of the XH001 LsrB and representative LsrB species: 1, XH001; 2, *S. typhimurium* 14028; 3, *E. coli* MG1655; 4, *A. actinomycetecomitans* HK1651; 5, and *Bacillus anthracis*. Alignment was conducted using MEGA7 (Kumar et al., 2016) via ClustalW Omega alignment method (Sievers et al., 2011). The conserved AA residues required for AI-2 binding are highlighted in bold, purple (K35, D116, D166, Q167, P220, and A222) and the corresponding XH001 conserved residue in bold, red. **(B)** The evolutionary relationship of the LsrB protein among the representative taxa. The evolutionary history was inferred by using the maximum-likelihood method based on the JTT matrix-based model (Jones et al., 1992). The tree with the highest log likelihood (-7027.33) is shown. Initial tree(s) for the heuristic search were obtained automatically by applying Neighbor-Join and BioNJ algorithms to a matrix of pairwise distances estimated using a JTT model, and then selecting the topology with superior log-likelihood value. The tree is drawn to scale, with branch lengths measured in the number of substitutions per site. The analysis involved 26 amino acid sequences. All positions containing gaps and missing data were masked. There were a total of 284 positions in the final dataset. Evolutionary analyses were conducted in MEGA7 (Kumar et al. (2016). **(C)** Protein sequence alignment of the XH001 LuxS protein and representative species. Marked in bold, yellow are the requisite amino acid residues (H,H, and C) that constitute the catalytic center of LuxS (Hilgers and Ludwig, 2001) and coordinate the Zn^2+^ ion: 1, XH001; 2, *S. typhimurium* 14028; 3, *E. coli* MG1655; 4, *A. actinomycetecomitans* HK1651; 5, *S. mutans* UA159. **(D)** The evolutionary relationship of the luxS protein among the representative taxa. The tree was generated using the same method parameters as **(B)**. The tree with the highest log likelihood (-1292.94) is shown. The analysis involved 5 amino acid sequences and there were a total of 157 positions in the final dataset.

Our recent transcriptomic analysis revealed the upregulation of more than 70 XH001 genes in excess of threefold when XH001 was physically associated with TM7x (He et al., 2015). APY09_02520, the first gene in a six-gene operon (**Supplementary Figure 1**), was most highly upregulated (∼20-fold), while the remaining genes in the same operon ranged from two to fivefold increases in transcription. NCBI-Blastp analysis annotated the predicted APY09_02520-encoded protein as an ortholog of LsrB, the receptor for binding the AI-2 QS molecule. Additionally, PHYRE 3-D structure folding analyses indicated the best hit in functional prediction of APY09_02520 as a putative AI-2 receptor (**Supplementary Data Sheet 1**). Thus, gene APY09_02520 was annotated as *lsrB* ortholog. However, contrary to the PHYRE analysis, protein sequence alignment revealed that XH001 LsrB shared low sequence identity with known representative LsrB species in *S. typhimurium* 14028 (23%), *E. coli* K-12 (26%), and *A. actinomycetecomitans* HK1651 (25%). Among the six proposed conserved amino acid residues (K35, D116, D166, Q167, P220, and A222) for identifying LsrB-like orthologs (Pereira et al., 2009), which are predicted to form hydrogen bonds with [(2*R*,4*S*)-2-methyl-2,3,3,4-tetrahydroxytetrahydrofuran (*R*THMF)], the AI-2 signaling molecule bound by the LsrB periplasmic binding protein in *S. typhimurium* (Miller et al., 2004), only Lysine at position 35 was identified in XH001 via protein sequence alignment (**Figure 1A**). To reconcile predictions from the two analysis methods, phylogenetic analyses, however, showed that XH001 LsrB is more evolutionarily related to AI-2 binding proteins, including LuxP, LsrB and RbsB, identified in *V. harveyi*, *S. typhimurium* 14028 as well as *E. coli*, and *A. actinomycetecommitans*, respectively (**Figure 1B**). Downstream of *lsrB*, the order of predicted genes within the same operon (**Supplementary Figure 1**) is as follows: *rbsA* (encoding Ribose ABC Transporter, ATP binding cassette), two *rbsC* (encoding Ribose ABC Transporter, permease protein) structural genes, and *dak1* gene that form a functional putative dihydroxy acetone kinase. Upstream of the putative *lsrB* ortholog, in the reverse direction, is annotated as a putative DeoR family transcriptional regulator for rhamnose utilization.

In addition to identifying the *lsrB* ortholog, genome analyses revealed that the XH001 genome also contains a *luxS* ortholog which encodes for an AI-2 synthase. Protein sequence analysis was employed to locate and confirm the previously determined requisite conserved amino acid residues (H, H, and C) that constitute the catalytic center of the LuxS protein and coordinate the Zn^2+^ ion (**Figure 1C**) (Hilgers and Ludwig, 2001). Phylogenetic analyses also determined its evolutionary relationship to other representative LuxS protein species (**Figure 1D**).

### Genetic System Development in XH001

**FIGURE 2:**
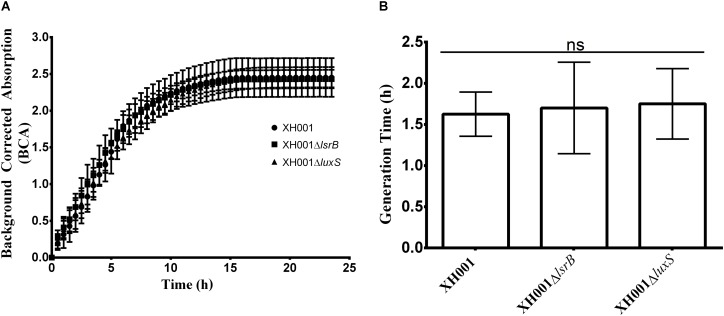
Representative growth kinetics and generation time analyses comparing wild type XH001, XH001Δ*lsrB*, and XH001Δ*luxS*. Wild type XH001, XH001Δ*lsrB*, and XH001Δ*luxS* were grown as indicated in methods for 24 hours, acquiring measurements (in the form of Background Corrected Absorption Units) every 30 minutes which produced a growth kinetic curve **(A)** and enabled quantification of generation time **(B)**. Each bar represents the average of three independent cultures performed in triplicate (*error bars*, SD) in two separate experiments. Statistical analysis was performed using one-way ANOVA.

Prior to this study, no genetic tools have been developed for either XH001 or TM7x. Since TM7x can currently not be cultivated in the absence of its XH001 host, which presents a major obstacle for genetic system development, we focused on XH001 instead. A genetic system was established for XH001 by adapting previous work by Yeung and Kozelsky (1994, 1997) on transformation of *Actinomyces* species (Yeung, 1995; Kolenbrander, 2000). To demonstrate transformability of XH001, pJRD215, a known broad-host range expression vector used in genetic studies of other closely-related *Actinomyces* species was electroporated into XH001 with similar transformation efficiencies previously reported in other *Actinomyces* species (Yeung and Kozelsky, 1994; Yeung, 1995). Utilizing the Km^R^ cassette, present in the pJRD215 plasmid, a chromosomal gene deletion construct was generated (see methods) to inactivate the *lsrB* and *luxS* genes in XH001 via homologous recombination (**Supplementary Figure 2**). Using a modified electroporation protocol, *lsrB* (XH001Δ*lsrB*) and *luxS* (XH001Δ*luxS*) gene deletion mutants were generated, albeit with low efficiencies compared to previous reports in other *Actinomyces* species (Yeung, 1995), which demonstrated that XH001 is genetically tractable. Gene deletion mutants were plated on kanamycin selective plates and were confirmed through sequencing. No growth defect was detected in either of the XH001Δ*lsrB* and XH001Δ*luxS* mutant backgrounds compared to wild type XH001 (**Figure 2**). Furthermore, neither XH001Δ*lsrB* nor XH001Δ*luxS* mutants displayed a discernable defect in TM7x association (**Supplementary Figure 3**) when tested using the established TM7x infection assay (He et al., 2015).

### TM7x Association Upregulates the Expression of *lsrB* in XH001 in a *luxS*-Dependent Manner

**FIGURE 3:**
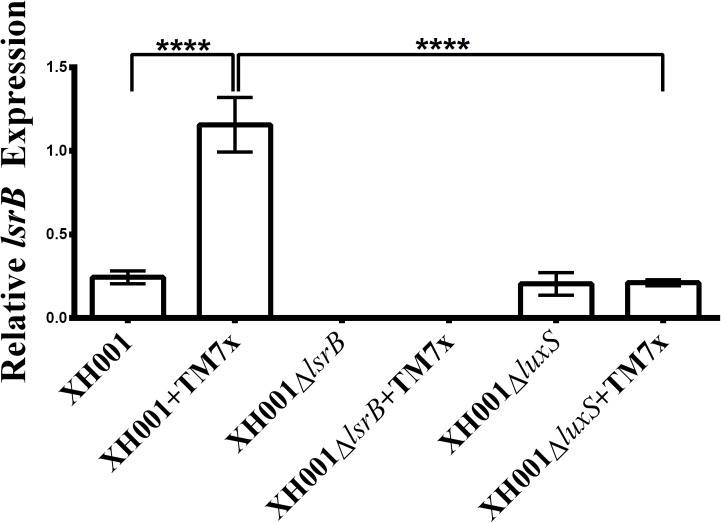
Quantification of *lsrB* expression. *lsrB* expression levels were monitored and each bar represents the average of two independent cultures performed in triplicate (*error bars*, SD). Significance is indicated when *p* < 0.0001 with four asterisks. Statistical analysis was performed using the Student’s *t*-test (two-tailed).

qRT-PCR analysis was used to quantify *lsrB* expression in the newly constructed mutant strains. TM7-associated XH001Δ*lsrB* and XH001Δ*lsrB* monoculture produced no *lsrB* transcripts as expected (**Figure 3**). Moreover, these results validated the initial transcriptomic profile, which revealed a statistically significant, 4.7-fold increase in *lsrB* expression in the TM7x-associated XH001 background compared to XH001 monoculture. Interestingly, while XH001Δ*luxS* displayed similar *lsrB* expression level as the XH001, the association of TM7x failed to induce enhanced *lsrB* expression, suggesting that TM7x-induced *lsrB* upregulation required the presence of *luxS* in XH001.

### Quantification of the AI-2 Signaling Molecule via GC-MS

**FIGURE 4:**
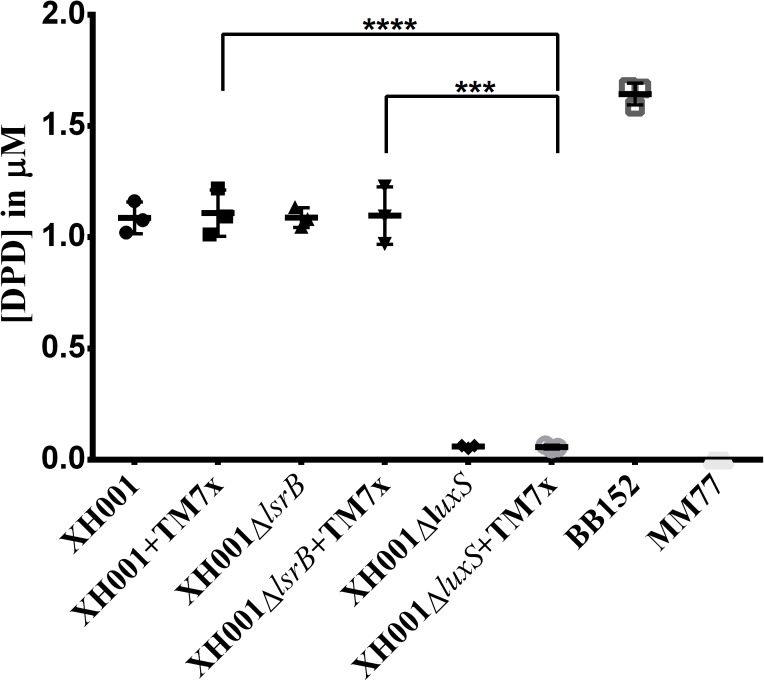
Quantification of the AI-2 signaling molecule via GC-MS. DPD concentration in media was evaluated. Each bar represents the average of one independent culture performed in triplicate (*error bars*, SD). Significance is indicated when *p* = 0.0002 with three asterisks and when *p* < 0.0001 with four asterisks.

It was of high interest to determine if XH001 indeed produces the AI-2 signaling molecule in a *luxS* dependent manner. GC-MS was employed to quantify DPD (Thiel et al., 2009), the precursor that randomly cyclizes into the group of equilibrium-connected isomers exclusively known as AI-2 autoinducers (Chen et al., 2002; Xavier et al., 2007), in the cell-free supernatants of XH001, XH001Δ*lsrB*, and XH001Δ*luxS* as monoculture, as well as their respective cocultures associated with TM7x. GC-MS quantification revealed that similar concentrations (∼1.1 μm) of DPD, were detected in the supernatant of both XH001 and XH001Δ*lsrB*, in monoculture and in TM7x-associated states, in quantities lower than an AI-2 signal-producing positive control, BB157, a *Vibrio harveyi* strain (**Figure 4**). Meanwhile, the *luxS* deletion resulted in abrogated DPD production independent of TM7x-association (**Figure 4**).

### Association of TM7x Enhances XH001 Biofilm Formation in a *lsrB*- and *luxS*-Dependent Manner

**FIGURE 5:**
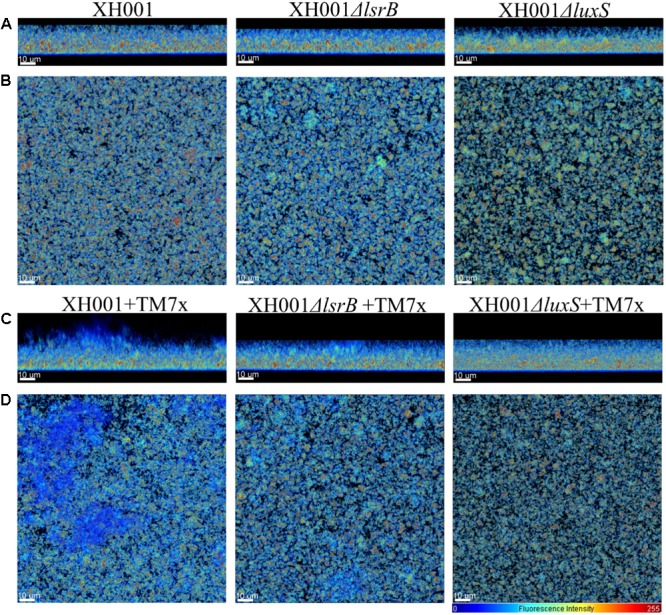
Representative 3D reconstructions of biofilms. Biofilms of wild type TM7x associated XH001, XH001Δ*lsrB*, *and* XH001Δ*luxS* monoculture as well as co-culture when they were associated with TM7x were grown (biological triplicate) as described in the Section “Materials and Methods.” Images were obtained by CLSM and reconstructed using Bitplane: Imaris-Microscopy Image Analysis Software (Bitplane). Reconstructed images led to a view of the same biofilms revealing a significant increase in overall biofilm thickness (height) in the TM7x-associated XH001 relative to XH001. **(A,C)** and **(B,D)** represent sagittal (xz) and horizontal (xy) projected images, respectively. The relative fluorescence intensity of the pseudo-colored images is reflected by the scale located in the lower right corner. All scale bars are 10 μm in length.

**FIGURE 6:**
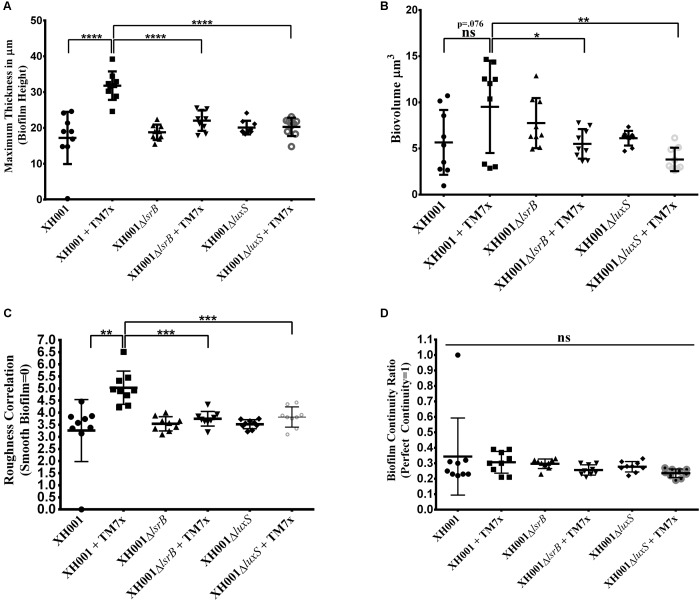
Quantification of total maximal biofilm thickness (height), biovolume, biofilm roughness correlation and biofilm continuity ratio. Histograms represents **(A)** Maximum Biofilm Thickness (Height), **(B)** Biovolume, **(C)** Biofilm Roughness Correlation, and **(D)** Biofilm Continuity Ratio, comparing wild type XH001, XH001Δ*lsrB*, and XH001Δ*luxS* monoculture as well as co-culture when they were associated with TM7x. The images analyzed were acquired with the same settings as indicated in this figure and in section “Materials and Methods”. Three independent cultures for each group were selected with 3 representative images between each culture. Each bar represents the average of two independent cultures performed in triplicate (*error bars*, SD). Significance is indicated when *p* < 0.05, *p* < 0.01, *p* < 0.001, and *p* < 0.0001 with one, two, three, and four asterisks, respectively. Statistical analysis was performed using the Student *t*-test (two tailed) and one-way ANOVA analysis using Graph Pad Prism 6 software.

AI-2 QS is widely studied and implicated as a universal signaling molecule in interspecies communication during multispecies oral biofilm development and in relation to the oral cavity influencing dental caries formation and periodontitis (Kolenbrander, 1997; Frias et al., 2001; Palmer et al., 2003; Kolenbrander et al., 2010). To test whether the XH001 encoded AI-2 QS system is involved in dual-species biofilm formation comprising of XH001 and its parasitic epibiont, TM7x, CLSM was utilized to visualize biofilm formation of XH001 wild type, Δ*lsrB* and Δ*luxS* mutant monocultures, as well as their respective co-cultures with TM7x. Confocal analysis showed that TM7x-associated XH001 formed a significantly thicker biofilm in height (31.79 μm) relative to XH001 (19.31 μm) as monospecies. Moreover, while XH001Δ*lsrB* (18.77 μm) and XH001Δ*luxS* (20.11 μm) did not display a biofilm deficit relative to XH001 wild type (19.31 μm) as monoculture, the association with TM7x failed to enhance biofilm formation in the *lsrB* (22.01 μm) and *luxS* (20.28 μm) mutant backgrounds (**Figures 5**, **6A**). Quantitative measurement further evaluated the biovolume, biofilm roughness correlation, and biofilm continuity ratio to demonstrate that TM7x induction of biofilm formation enhancement was present in the XH001 background, but not in the *lsrB* and *luxS* mutant backgrounds (**Figures 6B–D**). Specifically, TM7x-associated XH001 showed consistently an apparent higher biovolume (12.74 μm^3^), albeit this was not-statistically significant (*p* = 0.076), than XH001 monoculture (6.25 μm^3^) (**Figure 6B**). Meanwhile, the biofilm formation of TM7x-associated XH001 displayed significantly higher biovolume compared to that of TM7x-assocated *lsrB* or *luxS* mutant (**Figure 6B**). Furthermore, we observed a statistically significant increase in roughness correlation (structured smoothness of the biofilm) (*p* < 0.0001) in the TM7x-associated XH001 coculture background compared with XH001 as monoculture, while no such increase was observed in *lsrB* and *luxS* mutant backgrounds (**Figures 6A,C**). No statistically significant change in biofilm continuity ratio between any of the groups was observed (**Figure 6D**).

## Discussion

Epibiotic-parasitic relationships are predicted to be widespread among the recently discovered CPR group of ultra-small bacteria. The co-isolation and co-cultivation of TM7x, with its bacterial host, *A. odontolytics* subspecies *actinosynbacter* strain, XH001, has enabled the first characterization of a living Saccharibacteria organism, as well as the relationship between the two bacterial species. Our genetic and phenotypic analyses presented here strongly indicate an important role for the XH001 AI-2 QS system in modulating the epibiotic-parasitic relationship between XH001 and TM7x. Targeted transcriptional analysis via qRT-PCR in this study confirmed our prior findings that TM7x association triggered significant induction of gene APY09_02520 in XH001. Despite sharing low amino acid sequence identity with known LsrB species, NCBI Conserved Domain Structure Search, PHYRE2, as well as phylogenetic analyses all predicted that APY09_02520 encodes a putative LsrB ortholog which likely functions as receptor for the AI-2 signaling molecule. Thus, gene APY09_02520 was designated as a *lsrB* ortholog. NCBI-Blastp and protein sequence alignment analyses also revealed the presence of a *luxS* ortholog (**Figure 1**), the AI-2 QS signaling molecule synthase, in the XH001 genome. The *luxS* in XH001 was confirmed as an AI-2 synthase by GC-MS quantification, when a *luxS* deletion resulted in abrogated AI-2 signal production (**Figure 4**). These data, along with the observation that TM7x association induced upregulation of *lsrB* was dependent on *luxS* (**Figure 3**), strongly suggested that AI-2 QS is involved in XH001 and TM7x association. Furthermore, deletion of *luxS* significantly reduced the TM7x-association induced biofilm enhancement in XH001, suggesting that AI-2 QS is involved in regulating dual-species biofilm formation between XH001 and TM7x (**Figures 5**, **6**). Additional data supporting that *lsrB* is an AI-2 receptor and part of the AI-2 QS system was indicated by the requirement of both *lsrB* and *luxS* genes for TM7x association induced biofilm enhancement in wild type (**Figure 6**).

AI-2 signaling is well documented in regulating many bacterial interspecies interactions during biofilm formation, particularly in the oral cavity (Cuadra-Saenz et al., 2012). The development of these multispecies biofilms involves close physical interactions between bacteria and results in an intricate structural hierarchy enabling a complex avenue of bacterial communication, ultimately affecting the diffusible signal exchange efficiency (Kolenbrander et al., 2010). For example, oral *Streptococci* and *Actinomyces* are known to co-aggregate *in vitro* and in the natural environment being among the first to colonize the enamel by binding to the salivary pellicle. *Streptococcus oralis 34* and *Actinomyces naeslundii* T14V are incapable of forming monoculture biofilms, but form robust dual species biofilms when coaggregated (Palmer et al., 2003), which are mediated through *luxS* expression encoded in *S. oralis* (Rickard et al., 2006). Thus, the abolished increased dual-species biofilm formation observed in TM7x-associated XH001Δ*luxS* relative to TM7x-associated XH001 is consistent with previous reports.

Our bioinformatic analysis suggested that the QS system in XH001 is unlikely a canonical AI-2 signaling system. Previously, organisms with *lsrB*-like orthologs have been organized into two groups: > 60% *lsrB* protein sequence identity, a complete set of orthologs to the *lsr* genes, and presence of the six conserved amino acid residues in the binding pocket were considered a Group I member. If organisms are missing two or more orthologs of the *lsr* genes as well as at least two of the 6 residues of the AI-2 binding pocket, and exhibit < 60% protein sequence identity to LsrB in *S. typhimurium*, they were placed in Group II and not considered to have true AI-2 receptors (Pereira et al., 2009). The following bioinformatic criteria were established for determining potential *lsrB*-like orthologs: six conserved amino acid residues (K35, D116, D166, Q167, P220, and A222) were predicted to form hydrogen bonds with the AI-2 signaling molecule bound by the LsrB periplasmic binding protein in *S. typhimurium* (Miller et al., 2004; Pereira et al., 2009). Specifically, of the six residues (K35, D116, D166, Q167, P220, and A222), aspartate 166 and alanine 222 residues were determined to be required for AI-2 binding (Pereira et al., 2009). The LsrB protein in XH001 lacks aspartate 166 and alanine 222, but the K35, Lysine, is conserved.

Importantly, within the top 10 hits generated by PHYRE 3-D structure folding analysis of LsrB, the second was listed as putative ribose receptor, RbsB (**Supplementary Data Sheet 1**) and coupled with the presence of an intact ribose ABC transporter machinery downstream of *lsrB* in XH001 indicated that the XH001 *lsrB* operon could also be involved in ribose transport. Interestingly, it has been shown that the ribose substrate binding protein, RbsB in *A. actinomycetecomitans* HK1651 has binding affinity, albeit lower than LsrB, for AI-2 (James et al., 2006) and may facilitate the internalization of AI-2 (Shao et al., 2007a). Furthermore, ribose has also been shown to inhibit AI-2 internalization by competitive inhibition of AI-2 binding, thereby may dictate the binding preference for ribose or AI-2, and consequently affecting biofilm formation (James et al., 2006; Liu et al., 2017). While *A. actinomycetecomitans* HK1651 has two separate *lsr* and *rbs* operons, it is conceivable that the data summarized demonstrates that the identified XH001 *lsrB* operon could encode dual functionality for importing AI-2 and ribose, an interesting feature that warrants further investigation.

Overall, the data from this study reveals that TM7x association with XH001 augments biofilm formation via AI-2 QS. To our knowledge, this is the first time it has been demonstrated that a CPR bacterial species can promote biofilm formation capability of its bacterial host. However, the current available methods enabling visualization of the biofilm formation does not distinguish between the two species nor quantify the ratio of TM7x to XH001, or any potential biofilm matrix components involved in the observed biofilm enhancement. While there is no change in initial attachment and infectivity of TM7x (**Supplementary Figure 3**), the captured biofilm formation data suggests disrupted regulation of genes required for biofilm development in TM7x-associated XH001Δ*lsrB* and XH001Δ*luxS* backgrounds. It is also worthwhile to discuss the relative fitness advantage of TM7x induced biofilm formation relative to the oral cavity and fundamental clinical ramifications of their interaction. One possible advantage for inducing biofilm formation in XH001 via QS would be to hinder the inflammation response *in vivo*. Previously, TM7x-associated XH001 relative to XH001 as monoculture was shown to decrease TNF-α expression in macrophages (He et al., 2015). This observation is consistent with previous literature demonstrating that biofilm forming bacteria hinder recognition of the immune system (Donlan and Costerton, 2002; Domenech et al., 2012, 2013). Other literature suggests that the biofilm matrix production is associated with a decrease in the pro-inflammatory response by decreasing TNF-α production (Thurlow et al., 2011). Although the biofilm promoting genes in TM7x-associated XH001 are not currently elucidated, our previous transcriptomic analysis provided insight by revealing an expression increase of a gene set, including those which encode choline binding proteins (CPBs) in XH001 (He et al., 2015). CBPs have been associated with biofilm formation, adherence, phagocytosis, evasion of the innate immunity, and invasion of eukaryotic host cells (Rosenow et al., 1997; Luo et al., 2005; Moscoso et al., 2006; Schommer et al., 2011; Agarwal et al., 2013). TM7x induction of these genes could provide evolutionary fitness, enabling better survival of its host, XH001, in the oral cavity. The involvement of AI-2 QS in XH001 regulating these genes warrants further investigation.

## Conclusion

A newly developed genetic system generated gene deletion mutants in key AI-2 QS related genes which revealed the important role of AI-2 QS in augmenting the biofilm formation of XH001 when it was associated with its epibiont, TM7x. As evidence accumulates that a massive scale of uncultured bacteria may be obligate participants in relationships like that of TM7x and XH001, it is imperative to understand their interaction and provide basic knowledge for the study of other uncultivated bacterial species. In-depth characterization of AI-2 signaling between XH001 and TM7x, particularly in the context of biofilm formation and the development in periodontitis, could yield great insight into CPR bacteria, as well as their role in the microbial communities that so drastically influence human health and disease.

## Author Contributions

JB, BB, AE, RL, JM, WS, and XH designed the research. JB, BB, LC, AE, JM, and XH performed the research. JB, BB, LC, AE, RL, JM, WS, and XH analyzed the data. JB, BB, AE, RL, JM, WS, and XH wrote the manuscript.

## Conflict of Interest Statement

WS is an employee of C3J Therapeutics, Inc., which has licensed technologies from University of California Regents that could be indirectly related to this research project. The remaining authors declare that the research was conducted in the absence of any commercial or financial relationships that could be construed as a potential conflict of interest.
