# Supplementary figures and images for: Quorum Sensing Modulates the Epibiotic-Parasitic Relationship Between Actinomyces odontolyticus and Its Saccharibacteria epibiont, a Nanosynbacter lyticus Strain, TM7x
Source: Front Microbiol. 2018 Sep 24;9:2049. doi: 10.3389/fmicb.2018.02049 (PMC6166536; doi:10.3389/fmicb.2018.02049)

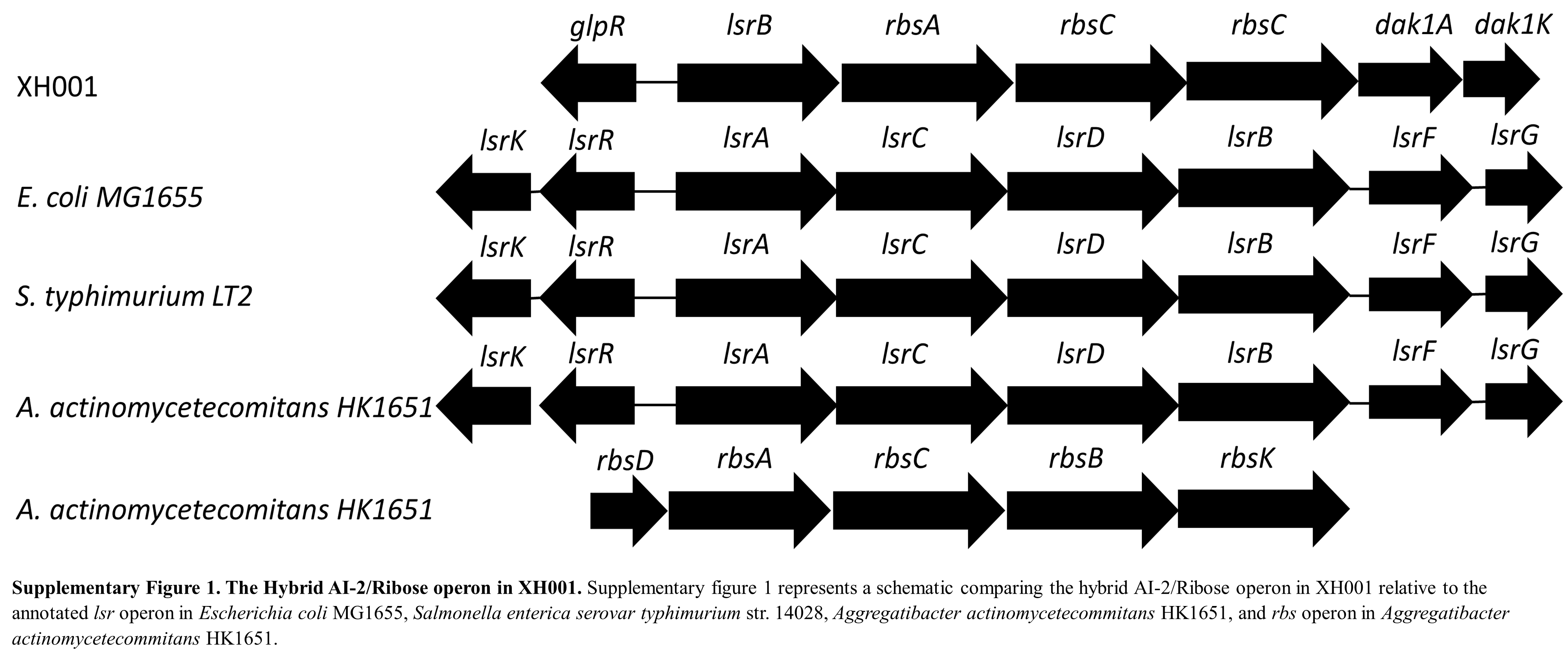

Supplement: Supplementary file 1 [file Image_1.tif]

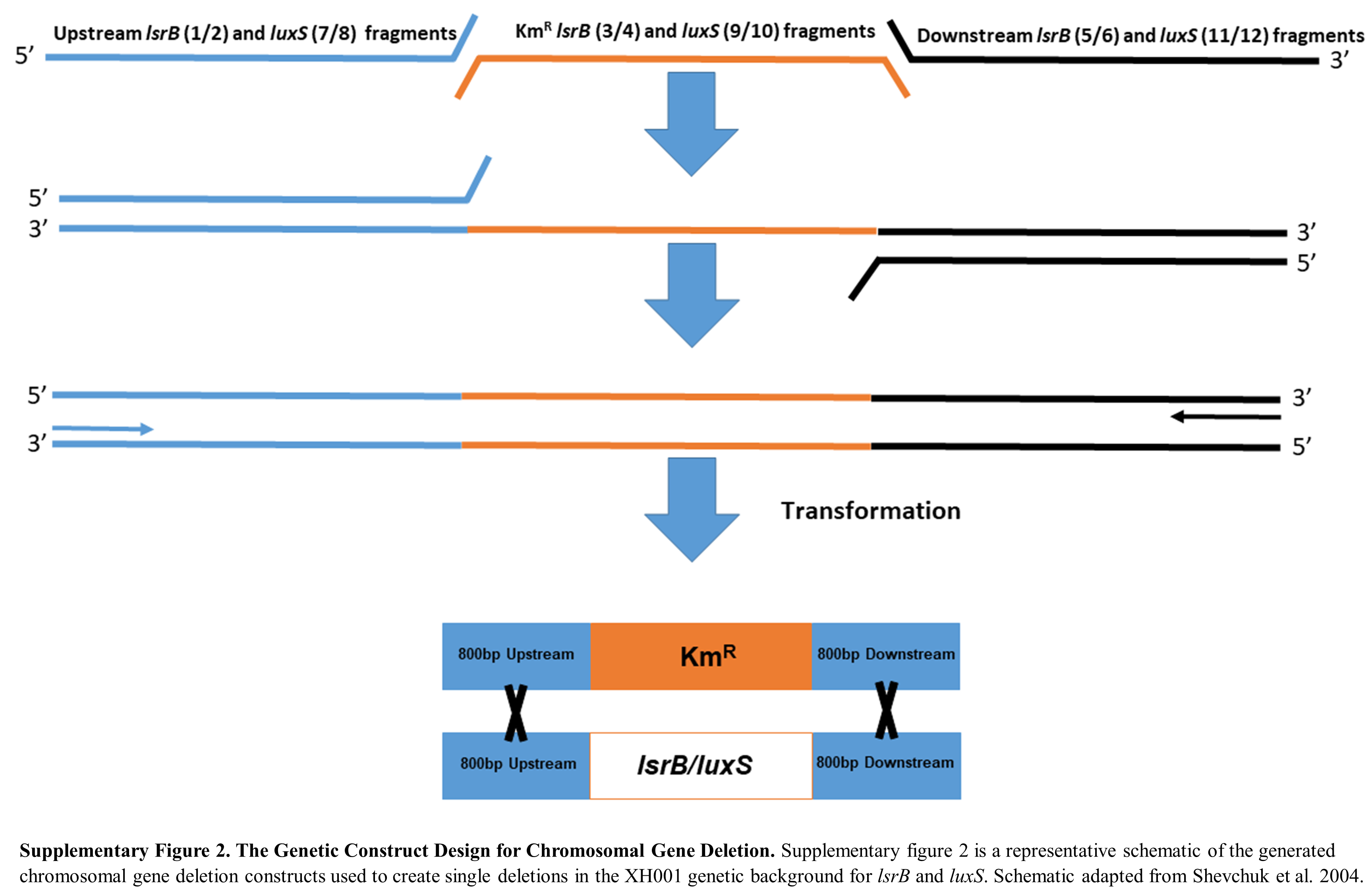

Supplement: Supplementary file 2 [file Image_2.tif]

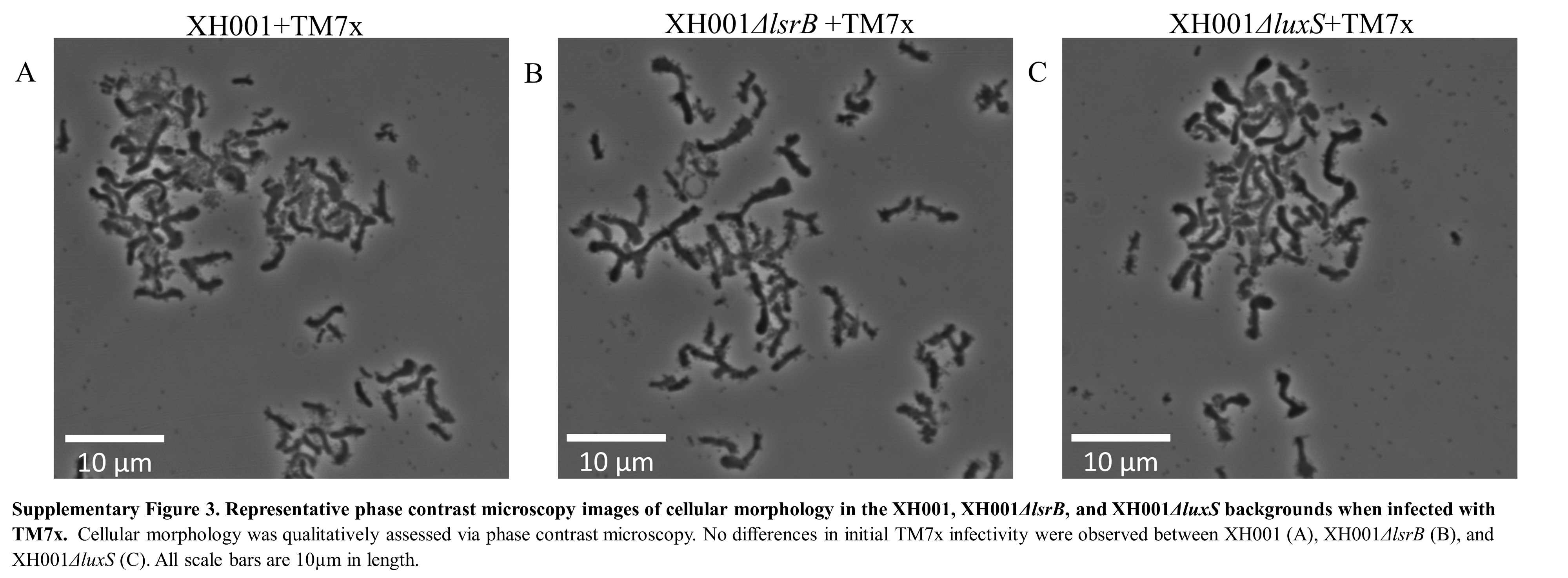

Supplement: Supplementary file 3 [file Image_3.tif]

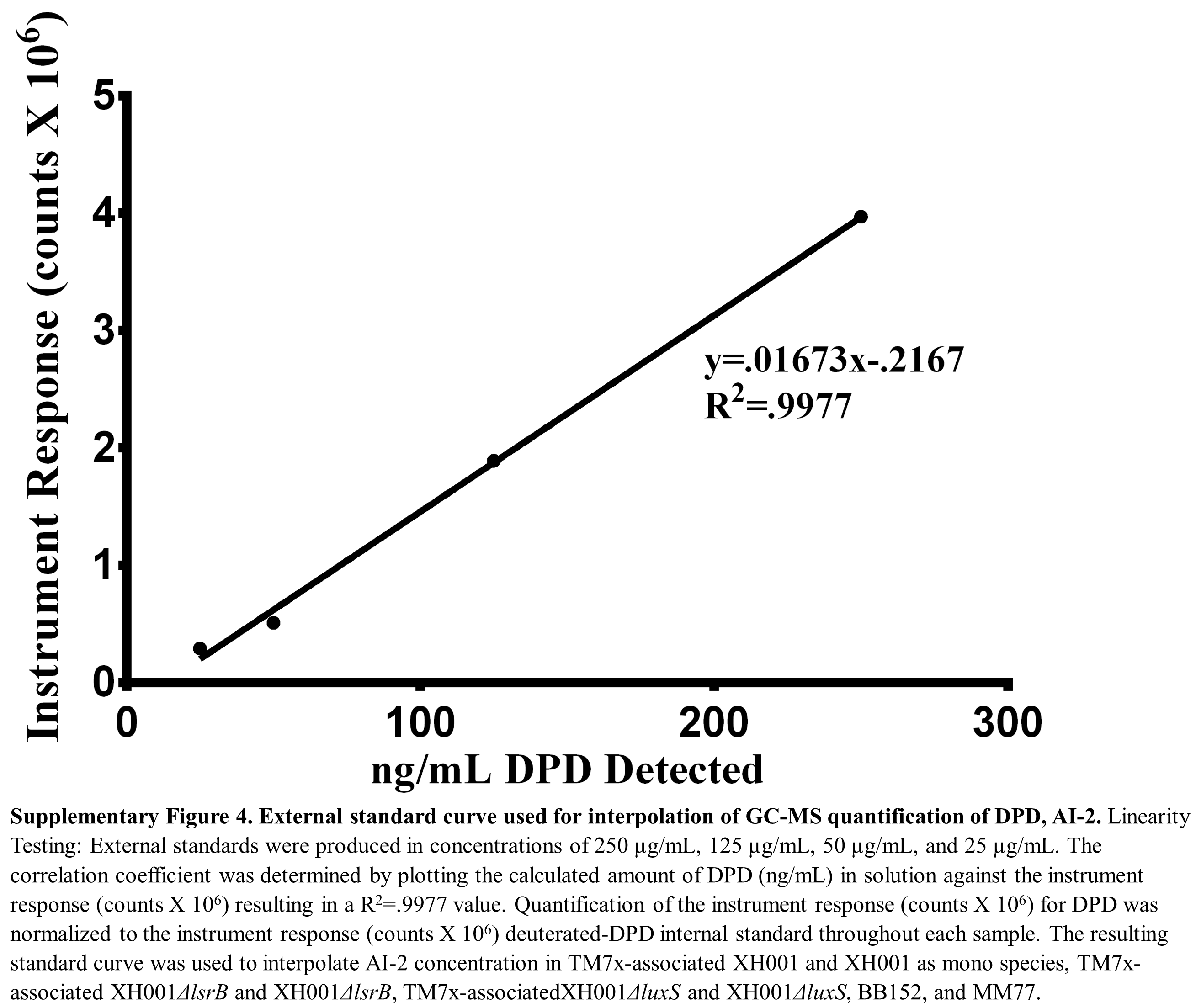

Supplement: Supplementary file 4 [file Image_4.tif]

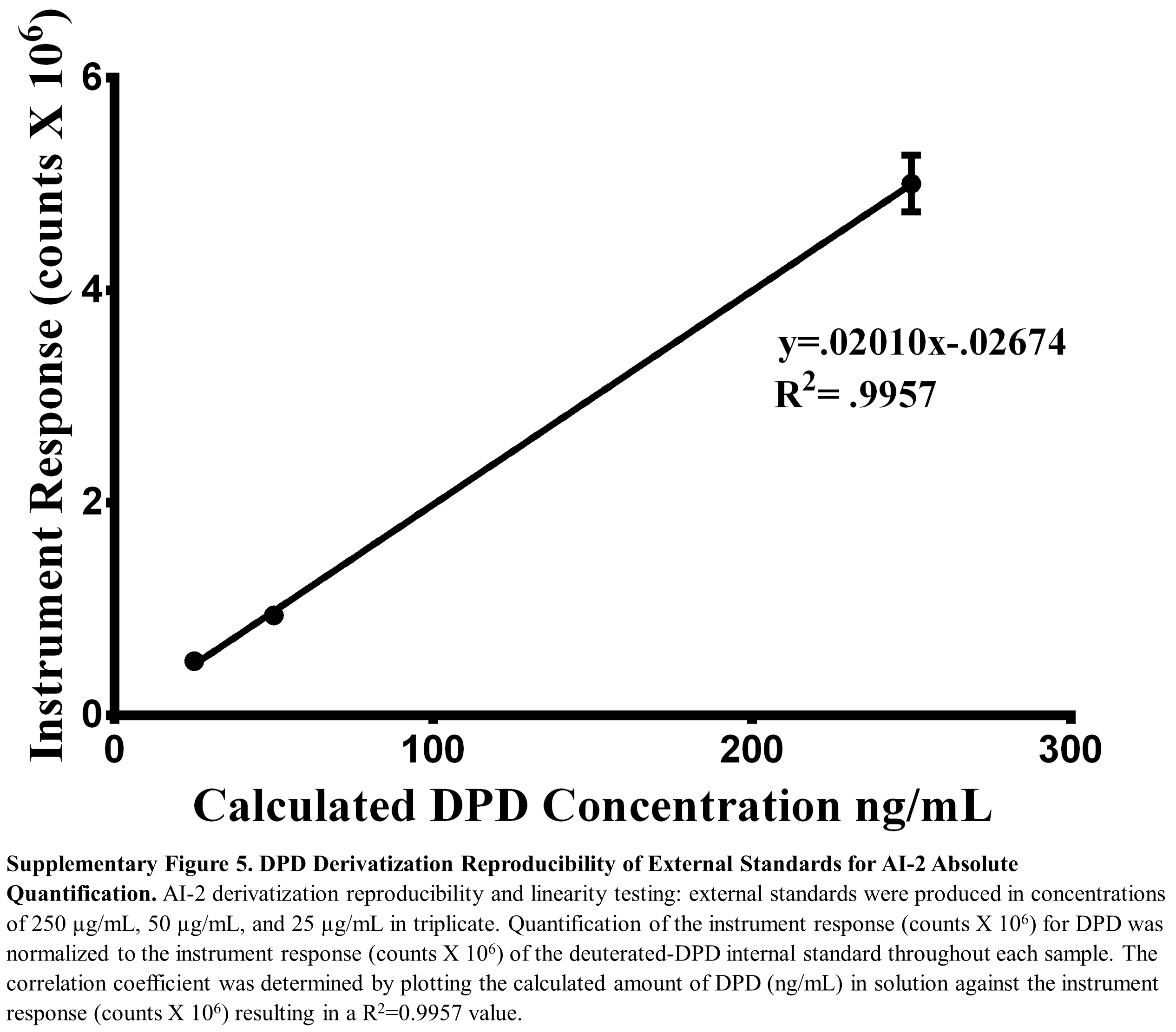

Supplement: Supplementary file 5 [file Image_5.tif]
