# Supplementary material for: Quorum Sensing Modulates the Epibiotic-Parasitic Relationship Between Actinomyces odontolyticus and Its Saccharibacteria epibiont, a Nanosynbacter lyticus Strain, TM7x
Source: Front Microbiol. 2018 Sep 24;9:2049. doi: 10.3389/fmicb.2018.02049 (PMC6166536; doi:10.3389/fmicb.2018.02049)
Supplement: Supplementary file 6 [file Table_1.docx]

| **Supplementary Table 1. Primer sequences used in this Study** | | |
| --- | --- | --- |
| **Primer Name/No.** | **Primer Sequence** | **Reference(s)** |
| XH001 *lsrB* F: | 5’-GGACGTCTCGCCGGAAAA-3’ | This Study |
| XH001 *lsrB* R: | 5’-CCTGTTCAAGGACGGAGGAA-3’ | This Study |
| F5 | 5’-GCGGAGCATGCGGATTA-3’ | ([Bor et al., 2016](#_ENREF_7)) |
| R3 | 5’-AACGTGCTGGCAACATAGGG-3’ | ([Bor et al., 2016](#_ENREF_7)) |
| 1 | 5’-CGAACACCAGGTTCAGGGAAGG-3’ | This Study |
| 2 | 5’-TGAGCGGGACTCTGGGGTTCGCGTCGTCACTTCCTCGTGATCTAG-3’ | This Study |
| 3 | 5’-CTAGATCACGAGGAAGTGACGACGCGAACCCCAGAGTCCCGCTCAG-3’ | This Study |
| 4 | 5’-GGCCGGGGCTGCGCGACCTGCAGCCAAGCTAGCTTCACG-3’ | This Study |
| 5 | 5’-GTGAAGCTAGCTTGGCTGCAGGTCGCGCAGCCCCGGCC-3’ | This Study |
| 6 | 5’-GTCCCGATCGCATGATGGTGA-3’ | This Study |
| 7 | 5’-TCCGCTCGGTCCTGTCTCAGAT-3’ | This Study |
| 8 | 5’-TGAGCGGGACTCTGGGGTTCGCCGGTTAGTCCCTTTCGGTGTAGTTTT-3’ | This Study |
| 9 | 5’-AAAACTACACCGAAAGGGACTAACCGGCGAACCCCAGAGTCCCGCTCA-3’ | This Study |
| 10 | 5’-CCAGGCGAACGCCCAGAACCTGCAGCCAAGCTAGCTTCAC-3’ | This Study |
| 11 | 5’-GTGAAGCTAGCTTGGCTGCAGGTTCTGGGCGTTCGCCTGG-3’ | This Study |
| 12 | 5’-TGGTTTTTCACAAATATGAGGCCAGA-3’ | This Study |
| 13 | 5’-cctaaatcgccagtccacag-3’ | This Study |
| 14 | 5’-GCTTGCCGAATATCATGGTG-3’ | This Study |
| 15 | 5’-cctaaatcgccagtccacag-3’ | This Study |
| 16 | 5’-gtcctcgtgtcgtatccattct-3’ | This Study |
| 17 | 5’-cacttcgtccaggtggtcct-3’ | This Study |
| 18 | 5’-CGAATATCATGGTGGAAAATGG-3’ | This Study |
| 19 | 5’-ctggcttcgaggagcttgac-3’ | This Study |
| 20 | 5’- gttaatgaggcgcacgtagg-3’ | This Study |
| 21 | 5’-ATGAAGATCGGAAGACTGACGGC-3’ | This Study |
| 22 | 5’-TCAGAAGTCGTAGTTCTTCGCTTGC-3’ | This Study |
| 23 | 5’-gagctttaccctggaccaca-3’ | This Study |
| 24 | 5’-agtggatgacgtcgacacg-3’ | This Study |
