# Supplementary material for: Quorum Sensing Modulates the Epibiotic-Parasitic Relationship Between Actinomyces odontolyticus and Its Saccharibacteria epibiont, a Nanosynbacter lyticus Strain, TM7x
Source: Front Microbiol. 2018 Sep 24;9:2049. doi: 10.3389/fmicb.2018.02049 (PMC6166536; doi:10.3389/fmicb.2018.02049)
Supplement: Supplementary file 7 [file Table_2.docx]

| **Supplementary Table 2. Gene Accession Numbers/Versions for the Proteins Analyzed in This Study** | | |
| --- | --- | --- |
| **Bacterial and Protein Species Name** | **Gene Accession No.** | **Version** |
| XH001 LsrB | KSW13249 | KSW13249.1 |
| *Escherichia coli* MG1655 LsrB | WP_000172465 | WP_000172465.1 |
| *Salmonella enterica serovar Typhimurium* ATCC 14028 LsrB | WP_000090737 | WP_000090737.1 |
| *Aggregatibacter actinomycetecommitans* HK1651 LsrB | AHN72389 | AHN72389.1 |
| *Bacillus anthracis* Strain Sterne | AJH47018 | AJH47018.1 |
| *Vibrio* *harveyi* LuxP | WP_012129029 | WP_012129029.1 |
| *Escherichia coli* MG1655 RbsB | WP_001056271 | WP_001056271.1 |
| *Salmonella enterica serovar Typhimurium* ATCC 14028 RbsB | WP_001056260 | WP_001056260.1 |
| *Aggregatibacter actinomycetecommitans* HK1651 RbsB | WP_005567919 | WP_005567919.1 |
| XH001 LuxS | WP_060566748 | WP_060566748.1 |
| *Escherichia coli* MG1655 LuxS | WP_001130211 | WP_001130211.1 |
| *Salmonella enterica serovar Typhimurium* ATCC 14028 LuxS | WP_001130194 | WP_001130194.1 |
| *Aggregatibacter actinomycetecommitans* HK1651 LuxS | WP_005548120 | WP_005548120.1 |
| *Streptococcus mutans* UA159 LuxS | WP_002263047 | WP_002263047.1 |
