# Supplementary material for: Quorum Sensing Modulates the Epibiotic-Parasitic Relationship Between Actinomyces odontolyticus and Its Saccharibacteria epibiont, a Nanosynbacter lyticus Strain, TM7x
Source: Front Microbiol. 2018 Sep 24;9:2049. doi: 10.3389/fmicb.2018.02049 (PMC6166536; doi:10.3389/fmicb.2018.02049)
Supplement: Supplementary file 8 [file Data_Sheet_1.PDF]

Phyre2

Email	jkbedree@ucla.edu
Description	lsrB_3D_structure_folding
Date	Thu Feb 8 16:11:22 GMT 2018
Unique Job ID	6cbb9278e8d95849

Detailed template information

#	Template	Alignment Coverage	3D Model	Confidence	% i.d.	Template Information
1	c4pz0A_	Alignment	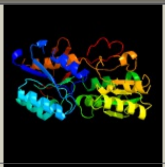	100.0	21	**PDB header:**sugar binding protein **Chain:** A: **PDB Molecule:**sugar abc transporter, sugar-binding protein; **PDBTitle:** the crystal structure of a solute binding protein from bacillus2 anthracis str. ames in complex with quorum-sensing signal3 autoinducer-2 (ai-2)
2	c5braA_	Alignment	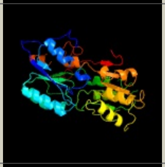	100.0	39	**PDB header:**solute-binding protein **Chain:** A: **PDB Molecule:**putative periplasmic binding protein with substrate ribose; **PDBTitle:** crystal structure of a putative periplasmic solute binding protein2 (ipr025997) from ochrobactrum anthropi atcc49188 (oant_2843, target3 efi-511085)
3	d1tjya_	Alignment	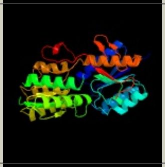	100.0	22	**Fold:**Periplasmic binding protein-like I **Superfamily:**Periplasmic binding protein-like I **Family:**L-arabinose binding protein-like
4	c4kvfA_	Alignment	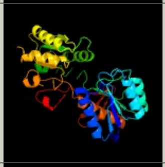	100.0	19	**PDB header:**transport protein **Chain:** A: **PDB Molecule:**rhamnose abc transporter, periplasmic rhamnose-binding **PDBTitle:** the crystal structure of a rhamnose abc transporter, periplasmic2 rhamnose-binding protein from kribbella flavida dsm 17836
5	c3d02A_	Alignment	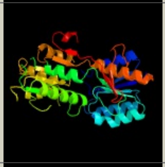	100.0	30	**PDB header:**sugar binding protein **Chain:** A: **PDB Molecule:**putative laci-type transcriptional regulator; **PDBTitle:** crystal structure of periplasmic sugar-binding protein2 (yp_001338366.1) from klebsiella pneumoniae subsp. pneumoniae mgh3 78578 at 1.30 a resolution
6	c4wzzA_	Alignment	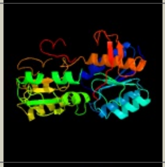	100.0	19	**PDB header:**transport protein **Chain:** A: **PDB Molecule:**putative sugar abc transporter, substrate-binding protein; **PDBTitle:** crystal structure of an abc transporter solute binding protein2 (ipr025997) from clostridium phytofermentas (cphy_0583, target efi-3 511148) with bound l-rhamnose
7	c5bq3A_	Alignment	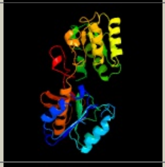	100.0	18	**PDB header:**transport protein **Chain:** A: **PDB Molecule:**rhamnose abc transporter, rhamnose-binding protein; **PDBTitle:** crystal structure of a sugar abc transporter (actodo_00688) from2 actinomyces odontolyticus atcc 17982 at 2.60 a resolution
8	c4y9tA_	Alignment	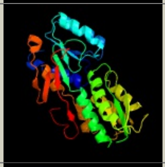	100.0	17	**PDB header:**solute-binding protein **Chain:** A: **PDB Molecule:**abc transporter, solute binding protein; **PDBTitle:** crystal structure of an abc transporter solute binding protein2 (ipr025997) from agrobacterium vitis s4 (avi_5305, target efi-511224)3 with bound alpha-d-glucosamine
9	c2rjoA_	Alignment	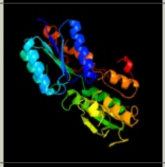	100.0	16	**PDB header:**signaling protein **Chain:** A: **PDB Molecule:**twin-arginine translocation pathway signal protein; **PDBTitle:** crystal structure of twin-arginine translocation pathway signal2 protein from burkholderia phytofirmans
10	c4wwhA_	Alignment	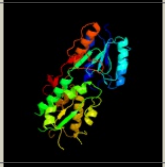	100.0	19	**PDB header:**transport protein **Chain:** A: **PDB Molecule:**abc transporter; **PDBTitle:** crystal structure of an abc transporter solute binding protein2 (ipr025997) from mycobacterium smegmatis (msmeg_1704, target efi-3 510967) with bound d-galactose
11	c3g1wB_	Alignment	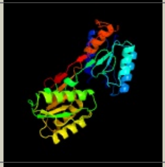	100.0	16	**PDB header:**transport protein **Chain:** B: **PDB Molecule:**sugar abc transporter; **PDBTitle:** crystal structure of sugar abc transporter (sugar-binding protein)2 from bacillus halodurans
			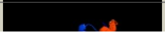			

12	c4rxuA_	Alignment	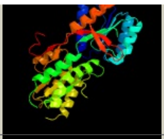	100.0	17	**PDB header:**transport protein<br>**Chain:** A: **PDB Molecule:**periplasmic sugar-binding protein;<br>**PDBTitle:** crystal structure of carbohydrate transporter solute binding protein2 caur_1924 from chloroflexus aurantiacus, target efi-511158, in3 complex with d-glucose
13	c3uugB_	Alignment	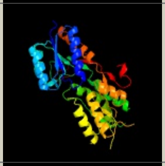	100.0	18	**PDB header:**sugar binding protein<br>**Chain:** B: **PDB Molecule:**multiple sugar-binding periplasmic receptor chve;<br>**PDBTitle:** crystal structure of the periplasmic sugar binding protein chve
14	c4pe6B_	Alignment	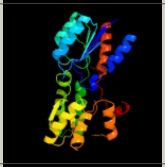	100.0	20	**PDB header:**solute-binding protein<br>**Chain:** B: **PDB Molecule:**putative abc transporter;<br>**PDBTitle:** crystal structure of abc transporter solute binding protein from2 thermobispora bispora dsm 43833
15	c4ys6A_	Alignment	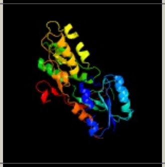	100.0	15	**PDB header:**solute-binding protein<br>**Chain:** A: **PDB Molecule:**putative solute-binding component of abc transporter;<br>**PDBTitle:** crystal structure of an abc transporter solute binding protein2 (ipr025997) from clostridium phytofermentans (cphy_1585, target efi-3 511156) with bound beta-d-glucose
16	c3h75A_	Alignment	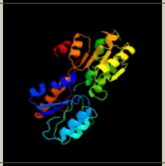	100.0	17	**PDB header:**sugar binding protein<br>**Chain:** A: **PDB Molecule:**periplasmic sugar-binding domain protein;<br>**PDBTitle:** crystal structure of a periplasmic sugar-binding protein from the2 pseudomonas fluorescens
17	c3ma0A_	Alignment	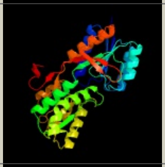	100.0	16	**PDB header:**sugar binding protein<br>**Chain:** A: **PDB Molecule:**d-xylose-binding periplasmic protein;<br>**PDBTitle:** closed liganded crystal structure of xylose binding protein from2 escherichia coli
18	c4z0nA_	Alignment	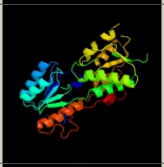	100.0	16	**PDB header:**transcription<br>**Chain:** A: **PDB Molecule:**periplasmic binding protein/laci transcriptional regulator;<br>**PDBTitle:** crystal structure of a periplasmic solute binding protein (ipr025997)2 from streptobacillus moniliformis dsm-12112 (smon_0317, target efi-3 511281) with bound d-galactose
19	c4rs3A_	Alignment	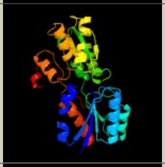	100.0	18	**PDB header:**transport protein<br>**Chain:** A: **PDB Molecule:**abc transporter, carbohydrate uptake transporter-2 (cut2)<br>**PDBTitle:** crystal structure of carbohydrate transporter a0qyb3 from2 mycobacterium smegmatis str. mc2 155, target efi-510969, in complex3 with xylitol
20	c5ix8A_	Alignment	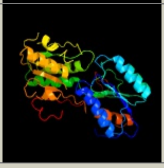	100.0	11	**PDB header:**transport protein<br>**Chain:** A: **PDB Molecule:**putative sugar abc transport system, substrate-binding<br>**PDBTitle:** crystal structure of sugar abc transport system, substrate-binding2 protein from bordetella parapertussis 12822
21	c5dkvD_	Alignment	not modelled	100.0	21	**PDB header:**sugar binding protein<br>**Chain:** D: **PDB Molecule:**abc transporter substrate binding protein (ribose);<br>**PDBTitle:** crystal structure of an abc transporter solute binding protein from2 agrobacterium vitis(avis_5339, target efi-511225) bound with alpha-d-3 tagatopyranose
22	c2qvcC_	Alignment	not modelled	100.0	20	**PDB header:**transport protein<br>**Chain:** C: **PDB Molecule:**sugar abc transporter, periplasmic sugar-binding protein;<br>**PDBTitle:** crystal structure of a periplasmic sugar abc transporter from2 thermotoga maritima
23	c3o1hB_	Alignment	not modelled	100.0	9	**PDB header:**signaling protein<br>**Chain:** B: **PDB Molecule:**periplasmic protein tort;<br>**PDBTitle:** crystal structure of the tors sensor domain - tort complex in the2 presence of tmao
24	c4yhsA_	Alignment	not modelled	100.0	15	**PDB header:**solute-binding protein<br>**Chain:** A: **PDB Molecule:**monosaccharide abc transporter substrate-binding protein,<br>**PDBTitle:** crystal structure of an abc transporter solute binding protein2 (ipr025997) from bradyrhizobium sp. btai1 (bbta_2440, target efi-3 511490) with bound bis-tris
25	c5hqjA_	Alignment	not modelled	100.0	19	**PDB header:**solute-binding protein<br>**Chain:** A: **PDB Molecule:**periplasmic binding protein/laci transcriptional regulator;<br>**PDBTitle:** crystal structure of abc transporter solute binding protein b1g1h72 from burkholderia graminis c4d1m, target efi-511179, in complex with3 d-arabinose
26	c2x7xA_	Alignment	not modelled	100.0	18	**PDB header:**transferase<br>**Chain:** A: **PDB Molecule:**sensor protein;<br>**PDBTitle:** fructose binding periplasmic domain of hybrid two component system2 bt1754
27	c4wutA_	Alignment	not modelled	100.0	19	**PDB header:**transport protein<br>**Chain:** A: **PDB Molecule:**abc transporter substrate binding protein (ribose);

27	c4wdtA_	Alignment	not modelled	100.0	19	**PDBTitle:** crystal structure of an abc transporter solute binding protein2 (ipr025997) from agrobacterium vitis (avi_5133, target efi-511220)3 with bound d-fucose
28	c4rxtA_	Alignment	not modelled	100.0	20	**PDB header:**transport protein<br>**Chain:** A: **PDB Molecule:**sugar abc transporter;<br>**PDBTitle:** crystal structure of carbohydrate transporter solute binding protein2 arad_9553 from agrobacterium radiobacter, target efi-511541, in3 complex with d-arabinose
29	d1jx6a_	Alignment	not modelled	100.0	12	**Fold:**Periplasmic binding protein-like I<br>**Superfamily:**Periplasmic binding protein-like I<br>**Family:**L-arabinose binding protein-like
30	c4ry8D_	Alignment	not modelled	100.0	16	**PDB header:**transport protein<br>**Chain:** D: **PDB Molecule:**periplasmic binding protein;<br>**PDBTitle:** crystal structure of 5-methylthioribose transporter solute binding2 protein tlet_1677 from thermotoga lettingae tmo target efi-511109 in3 complex with 5-methylthioribose
31	d1gcaa_	Alignment	not modelled	100.0	18	**Fold:**Periplasmic binding protein-like I<br>**Superfamily:**Periplasmic binding protein-like I<br>**Family:**L-arabinose binding protein-like
32	c4kq9A_	Alignment	not modelled	100.0	18	**PDB header:**transport protein<br>**Chain:** A: **PDB Molecule:**ribose abc transporter, substrate binding protein;<br>**PDBTitle:** crystal structure of periplasmic ribose abc transporter from2 conexibacter woesei dsm 14684
33	c4kqcA_	Alignment	not modelled	100.0	14	**PDB header:**transport protein<br>**Chain:** A: **PDB Molecule:**periplasmic binding protein/laci transcriptional regulator;<br>**PDBTitle:** abc transporter, laci family transcriptional regulator from2 brachyspira murdochii
34	c4ry9A_	Alignment	not modelled	100.0	18	**PDB header:**transport protein<br>**Chain:** A: **PDB Molecule:**periplasmic binding protein/laci transcriptional regulator;<br>**PDBTitle:** crystal structure of carbohydrate transporter solute binding protein2 veis_2079 from verminephrobacter eiseniae ef01-2, target efi-511009,3 a complex with d-talitol
35	c3ksmA_	Alignment	not modelled	100.0	21	**PDB header:**transport protein<br>**Chain:** A: **PDB Molecule:**abc-type sugar transport system, periplasmic component;<br>**PDBTitle:** crystal structure of abc-type sugar transport system, periplasmic2 component from hahella chejuensis
36	d2fvya1	Alignment	not modelled	100.0	17	**Fold:**Periplasmic binding protein-like I<br>**Superfamily:**Periplasmic binding protein-like I<br>**Family:**L-arabinose binding protein-like
37	c5hsgA_	Alignment	not modelled	100.0	17	**PDB header:**transport protein<br>**Chain:** A: **PDB Molecule:**putative abc transporter, nucleotide binding/atpase<br>**PDBTitle:** crystal structure of an abc transporter solute binding protein from2 klebsiella pneumoniae (kpn_01730, target efi-511059), apo open3 structure
38	c4irxA_	Alignment	not modelled	100.0	16	**PDB header:**transport protein<br>**Chain:** A: **PDB Molecule:**sugar abc transporter, periplasmic sugar-binding protein;<br>**PDBTitle:** crystal structure of caulobacter myo-inositol binding protein bound to2 myo-inositol
39	c4yleA_	Alignment	not modelled	100.0	20	**PDB header:**transport protein<br>**Chain:** A: **PDB Molecule:**periplasmic binding protein/laci transcriptional regulator;<br>**PDBTitle:** crystal structure of an abc transpoter solute binding protein2 (ipr025997) from burkholderia multivorans (bmul_1631, target efi-3 511115) with an unknown ligand modelled as alpha-d-erythrofuranose
40	c5dteD_	Alignment	not modelled	100.0	21	**PDB header:**transport protein<br>**Chain:** D: **PDB Molecule:**monosaccharide-transporting atpase;<br>**PDBTitle:** crystal structure of an abc transporter periplasmic solute binding2 protein (ipr025997) from actinobacillus succinogenes 130z(asuc_0081,3 target efi-511065) with bound d-allose
41	c4kzkA_	Alignment	not modelled	100.0	15	**PDB header:**sugar binding protein<br>**Chain:** A: **PDB Molecule:**l-arabinose abc transporter, periplasmic l-arabinose-<br>**PDBTitle:** the structure of the periplasmic l-arabinose binding protein from2 burkholderia thailandensis
42	d8abpa_	Alignment	not modelled	100.0	15	**Fold:**Periplasmic binding protein-like I<br>**Superfamily:**Periplasmic binding protein-like I<br>**Family:**L-arabinose binding protein-like
43	c5xsdA_	Alignment	not modelled	100.0	18	**PDB header:**sugar binding protein<br>**Chain:** A: **PDB Molecule:**periplasmic binding protein/laci transcriptional regulator;<br>**PDBTitle:** xylfii-lytsn complex mutant - d103a
44	c4yv7A_	Alignment	not modelled	100.0	19	**PDB header:**solute-binding protein<br>**Chain:** A: **PDB Molecule:**periplasmic binding protein/laci transcriptional regulator;<br>**PDBTitle:** crystal structure of an abc transporter solute binding protein2 (ipr025997) from mycobacterium smegmatis (msmei_3018, target efi-3 511327) with bound glycerol
45	c4joqA_	Alignment	not modelled	100.0	19	**PDB header:**transport protein<br>**Chain:** A: **PDB Molecule:**abc ribose transporter, periplasmic solute-binding protein;<br>**PDBTitle:** putative ribose abc transporter, periplasmic solute-binding protein2 from rhodobacter sphaeroides
46	c3gbvB_	Alignment	not modelled	100.0	12	**PDB header:**transcription regulator<br>**Chain:** B: **PDB Molecule:**putative laci-family transcriptional regulator;<br>**PDBTitle:** crystal structure of a putative laci transcriptional regulator from2 bacteroides fragilis
47	c4wt7B_	Alignment	not modelled	100.0	20	**PDB header:**transport protein<br>**Chain:** B: **PDB Molecule:**abc transporter substrate binding protein (ribose);<br>**PDBTitle:** crystal structure of an abc transporter solute binding protein2 (ipr025997) from agrobacterium vitis (avi_5165, target efi-511223)3 with bound allitol

48	c3brsA_	Alignment	not modelled	100.0	20	**PDB header:**transport protein<br>**Chain:** A: **PDB Molecule:**periplasmic binding protein/laci transcriptional regulator;<br>**PDBTitle:** crystal structure of sugar transporter from clostridium2 phytofermentans
49	c4rxmA_	Alignment	not modelled	100.0	12	**PDB header:**transport protein<br>**Chain:** A: **PDB Molecule:**possible sugar abc superfamily atp binding cassette<br>**PDBTitle:** crystal structure of periplasmic abc transporter solute binding2 protein a7jw62 from mannheimia haemolytica phl213, target efi-511105,3 in complex with myo-inositol
50	c3rotA_	Alignment	not modelled	100.0	12	**PDB header:**transport protein<br>**Chain:** A: **PDB Molecule:**abc sugar transporter, periplasmic sugar binding protein;<br>**PDBTitle:** crystal structure of abc sugar transporter (periplasmic sugar binding2 protein) from legionella pneumophila
51	c4yo7A_	Alignment	not modelled	100.0	15	**PDB header:**solute binding protein<br>**Chain:** A: **PDB Molecule:**sugar abc transporter (sugar-binding protein);<br>**PDBTitle:** crystal structure of an abc transporter solute binding protein2 (ipr025997) from bacillus halodurans c-125 (bh2323, target efi-3 511484) with bound myo-inositol
52	d1guda_	Alignment	not modelled	100.0	18	**Fold:**Periplasmic binding protein-like I<br>**Superfamily:**Periplasmic binding protein-like I<br>**Family:**L-arabinose binding protein-like
53	c5jx2A_	Alignment	not modelled	100.0	16	**PDB header:**transport protein<br>**Chain:** A: **PDB Molecule:**glucose/galactose-binding lipoprotein;<br>**PDBTitle:** crystal structure of mglb-2 (tp0684) from treponema pallidum
54	c4ry0A_	Alignment	not modelled	100.0	18	**PDB header:**transport protein<br>**Chain:** A: **PDB Molecule:**probable ribose abc transporter, substrate-binding protein;<br>**PDBTitle:** crystal structure of ribose transporter solute binding protein2 rhe_pf00037 from rhizobium etli cfn 42, target efi-511357, in complex3 with d-ribose
55	c4ru1C_	Alignment	not modelled	100.0	16	**PDB header:**transport protein<br>**Chain:** C: **PDB Molecule:**monosaccharide abc transporter substrate-binding protein,<br>**PDBTitle:** crystal structure of carbohydrate transporter acei_1806 from2 acidothermus cellulolyticus 11b, target efi-510965, in complex with3 myo-inositol
56	c4rweA_	Alignment	not modelled	100.0	15	**PDB header:**sugar binding protein<br>**Chain:** A: **PDB Molecule:**sugar-binding transport protein;<br>**PDBTitle:** the crystal structure of a sugar-binding transport protein from2 yersinia pestis co92
57	c2vk2A_	Alignment	not modelled	100.0	19	**PDB header:**transport protein<br>**Chain:** A: **PDB Molecule:**abc transporter periplasmic-binding protein ytfq;<br>**PDBTitle:** crystal structure of a galactofuranose binding protein
58	c3l6uA_	Alignment	not modelled	100.0	15	**PDB header:**transport protein<br>**Chain:** A: **PDB Molecule:**abc-type sugar transport system periplasmic component;<br>**PDBTitle:** crystal structure of abc-type sugar transport system, periplasmic2 component from exiguobacterium sibiricum
59	c3l49D_	Alignment	not modelled	100.0	18	**PDB header:**transport protein<br>**Chain:** D: **PDB Molecule:**abc sugar (ribose) transporter, periplasmic substrate-<br>**PDBTitle:** crystal structure of abc sugar transporter subunit from rhodobacter2 sphaeroides 2.4.1
60	c2ioyB_	Alignment	not modelled	100.0	19	**PDB header:**sugar binding protein<br>**Chain:** B: **PDB Molecule:**periplasmic sugar-binding protein;<br>**PDBTitle:** crystal structure of thermoanaerobacter tengcongensis ribose binding2 protein
61	d2dria_	Alignment	not modelled	100.0	18	**Fold:**Periplasmic binding protein-like I<br>**Superfamily:**Periplasmic binding protein-like I<br>**Family:**L-arabinose binding protein-like
62	c2fn9A_	Alignment	not modelled	100.0	17	**PDB header:**sugar binding protein<br>**Chain:** A: **PDB Molecule:**ribose abc transporter, periplasmic ribose-binding protein;<br>**PDBTitle:** thermotoga maritima ribose binding protein unliganded form
63	c3h5oB_	Alignment	not modelled	100.0	12	**PDB header:**transcription regulator<br>**Chain:** B: **PDB Molecule:**transcriptional regulator gntr;<br>**PDBTitle:** the crystal structure of transcription regulator gntr from2 chromobacterium violaceum
64	c3d8uA_	Alignment	not modelled	100.0	11	**PDB header:**transcription regulator<br>**Chain:** A: **PDB Molecule:**purr transcriptional regulator;<br>**PDBTitle:** the crystal structure of a purr family transcriptional regulator from2 vibrio parahaemolyticus rimd 2210633
65	c2fqxA_	Alignment	not modelled	100.0	9	**PDB header:**transport protein<br>**Chain:** A: **PDB Molecule:**membrane lipoprotein tmpc;<br>**PDBTitle:** pnra from treponema pallidum complexed with guanosine
66	c2iksA_	Alignment	not modelled	100.0	9	**PDB header:**transcription<br>**Chain:** A: **PDB Molecule:**dna-binding transcriptional dual regulator;<br>**PDBTitle:** crystal structure of n-terminal truncated dna-binding transcriptional2 dual regulator from escherichia coli k12
67	c3e3mA_	Alignment	not modelled	100.0	14	**PDB header:**transcription<br>**Chain:** A: **PDB Molecule:**transcriptional regulator, laci family;<br>**PDBTitle:** crystal structure of a laci family transcriptional regulator from2 silicibacter pomeroyi
68	c3mizB_	Alignment	not modelled	100.0	14	**PDB header:**transcription regulator<br>**Chain:** B: **PDB Molecule:**putative transcriptional regulator protein, laci<br>**PDBTitle:** crystal structure of a putative transcriptional regulator2 protein, lacl family from rhizobium etli
69	c2rgyA_	Alignment	not modelled	100.0	14	**PDB header:**transcription regulator<br>**Chain:** A: **PDB Molecule:**transcriptional regulator, laci family;<br>**PDBTitle:** crystal structure of transcriptional regulator of laci family from2 burkhoderia phymatum
70	c3c3kA_	Alignment	not modelled	100.0	11	**PDB header:**isomerase<br>**Chain:** A: **PDB Molecule:**alanine racemase;

70	[illegible]	Alignment	not modelled	100.0	11	**PDBTitle:** crystal structure of an uncharacterized protein from actinobacillus2 succinogenes
71	c4o5aA_	Alignment	not modelled	100.0	10	**PDB header:**transcription regulator<br>**Chain:** A: **PDB Molecule:**laci family transcription regulator;<br>**PDBTitle:** the crystal structure of a laci family transcriptional regulator from2 bifidobacterium animalis subsp. lactis dsm 10140
72	c5er3A_	Alignment	not modelled	100.0	17	**PDB header:**solute-binding protein<br>**Chain:** A: **PDB Molecule:**sugar abc transporter, periplasmic sugar-binding protein;<br>**PDBTitle:** crystal structure of abc transporter system solute-binding protein2 from rhodopirellula baltica sh 1
73	c3k4hA_	Alignment	not modelled	100.0	11	**PDB header:**transcription regulator<br>**Chain:** A: **PDB Molecule:**putative transcriptional regulator;<br>**PDBTitle:** crystal structure of putative transcriptional regulator laci from2 bacillus cereus subsp. cytotoxis nvh 391-98
74	c4iilA_	Alignment	not modelled	100.0	11	**PDB header:**membrane protein<br>**Chain:** A: **PDB Molecule:**membrane lipoprotein tpn38(b);<br>**PDBTitle:** crystal structure of rfua (tp0298) of t. pallidum bound to riboflavin
75	c3hcwB_	Alignment	not modelled	100.0	9	**PDB header:**rna binding protein<br>**Chain:** B: **PDB Molecule:**maltose operon transcriptional repressor;<br>**PDBTitle:** crystal structure of probable maltose operon transcriptional repressor2 malr from staphylococcus areus
76	c1jyeA_	Alignment	not modelled	100.0	12	**PDB header:**transcription<br>**Chain:** A: **PDB Molecule:**lactose operon repressor;<br>**PDBTitle:** structure of a dimeric lac repressor with c-terminal deletion and k84l2 substitution
77	d1jyea_	Alignment	not modelled	100.0	12	**Fold:**Periplasmic binding protein-like I<br>**Superfamily:**Periplasmic binding protein-like I<br>**Family:**L-arabinose binding protein-like
78	c3brqA_	Alignment	not modelled	100.0	16	**PDB header:**transcription regulator<br>**Chain:** A: **PDB Molecule:**hth-type transcriptional regulator ascg;<br>**PDBTitle:** crystal structure of the escherichia coli transcriptional repressor2 ascg
79	c3jy6B_	Alignment	not modelled	100.0	12	**PDB header:**transcription regulator<br>**Chain:** B: **PDB Molecule:**transcriptional regulator, laci family;<br>**PDBTitle:** crystal structure of laci transcriptional regulator from lactobacillus2 brevis
80	c3gv0A_	Alignment	not modelled	100.0	11	**PDB header:**transcription regulator<br>**Chain:** A: **PDB Molecule:**transcriptional regulator, laci family;<br>**PDBTitle:** crystal structure of laci family transcription regulator from2 agrobacterium tumefaciens
81	c3g85A_	Alignment	not modelled	100.0	9	**PDB header:**transcription regulator<br>**Chain:** A: **PDB Molecule:**transcriptional regulator (laci family);<br>**PDBTitle:** crystal structure of laci family transcription regulator from2 clostridium acetobutylicum
82	d2nzug1	Alignment	not modelled	100.0	10	**Fold:**Periplasmic binding protein-like I<br>**Superfamily:**Periplasmic binding protein-like I<br>**Family:**L-arabinose binding protein-like
83	c4rkqD_	Alignment	not modelled	100.0	13	**PDB header:**transcription regulator<br>**Chain:** D: **PDB Molecule:**transcriptional regulator, laci family;<br>**PDBTitle:** crystal structure of laci family transcriptional regulator from2 arthrobacter sp. fb24, target efi-560007
84	c3kkeA_	Alignment	not modelled	100.0	14	**PDB header:**transcription regulator<br>**Chain:** A: **PDB Molecule:**laci family transcriptional regulator;<br>**PDBTitle:** crystal structure of a laci family transcriptional regulator from2 mycobacterium smegmatis
85	c3qk7C_	Alignment	not modelled	100.0	15	**PDB header:**transcription regulator<br>**Chain:** C: **PDB Molecule:**transcriptional regulators;<br>**PDBTitle:** crystal structure of putative transcriptional regulator from yersinia2 pestis biovar microtus str. 91001
86	c3hs3A_	Alignment	not modelled	100.0	17	**PDB header:**transcription regulator<br>**Chain:** A: **PDB Molecule:**ribose operon repressor;<br>**PDBTitle:** crystal structure of periplasmic binding ribose operon2 repressor protein from lactobacillus acidophilus
87	c3egcF_	Alignment	not modelled	100.0	12	**PDB header:**structural genomics, unknown function<br>**Chain:** F: **PDB Molecule:**putative ribose operon repressor;<br>**PDBTitle:** crystal structure of a putative ribose operon repressor from2 burkholderia thailandensis
88	c3k9cA_	Alignment	not modelled	100.0	12	**PDB header:**transcription regulator<br>**Chain:** A: **PDB Molecule:**transcriptional regulator, laci family protein;<br>**PDBTitle:** crystal structure of laci transcriptional regulator from rhodococcus2 species.
89	c3dbiA_	Alignment	not modelled	100.0	17	**PDB header:**transcription regulator<br>**Chain:** A: **PDB Molecule:**sugar-binding transcriptional regulator, laci family;<br>**PDBTitle:** crystal structure of sugar-binding transcriptional regulator (laci2 family) from escherichia coli complexed with phosphate
90	c4rk1F_	Alignment	not modelled	100.0	12	**PDB header:**transcription regulator<br>**Chain:** F: **PDB Molecule:**ribose transcriptional regulator;<br>**PDBTitle:** crystal structure of laci family transcriptional regulator from2 enterococcus faecium, target efi-512930, with bound ribose
91	c2hqbA_	Alignment	not modelled	100.0	15	**PDB header:**transcription<br>**Chain:** A: **PDB Molecule:**transcriptional activator of comk gene;<br>**PDBTitle:** crystal structure of a transcriptional activator of comk2 gene from bacillus halodurans
92	c4rk5A_	Alignment	not modelled	100.0	13	**PDB header:**transcription regulator<br>**Chain:** A: **PDB Molecule:**transcriptional regulator, laci family;<br>**PDBTitle:** crystal structure of laci family transcriptional regulator from2 lactobacillus casei, target efi-512911, with bound sucrose
93	c3cs3A_	Alignment	not modelled	100.0	10	**PDB header:**transcription regulator<br>**Chain:** A: **PDB Molecule:**sugar-binding transcriptional regulator, laci family;<br>**PDBTitle:** crystal structure of sugar-binding transcriptional regulator (laci2 family) from enterococcus faecalis
94	c2qu7B_	Alignment	not modelled	100.0	13	**PDB header:**transcription<br>**Chain:** B: **PDB Molecule:**putative transcriptional regulator;<br>**PDBTitle:** crystal structure of a putative transcription regulator from2 staphylococcus saprophyticus subsp. saprophyticus
						**PDB header:**regulatory protein

95	c3bblA_	Alignment	not modelled	99.9	9	**Chain:** A: **PDB Molecule:**regulatory protein of laci family;<br>**PDBTitle:** crystal structure of a regulatory protein of laci family from2 chloroflexus aggregans
96	c4rk6B_	Alignment	not modelled	99.9	14	**PDB header:**transcription regulator<br>**Chain:** B: **PDB Molecule:**glucose-resistance amylase regulator;<br>**PDBTitle:** crystal structure of laci family transcriptional regulator ccpa from2 weissella paramesenteroides, target efi-512926, with bound glucose
97	c3ctpB_	Alignment	not modelled	99.9	16	**PDB header:**transcription regulator<br>**Chain:** B: **PDB Molecule:**periplasmic binding protein/laci transcriptional regulator;<br>**PDBTitle:** crystal structure of periplasmic binding protein/laci transcriptional2 regulator from alkaliphilus metalliredigens qymf complexed with d-3 xylulofuranose
98	d1tlfa_	Alignment	not modelled	99.9	12	**Fold:**Periplasmic binding protein-like I<br>**Superfamily:**Periplasmic binding protein-like I<br>**Family:**L-arabinose binding protein-like
99	c5ufhA_	Alignment	not modelled	99.9	10	**PDB header:**transcription<br>**Chain:** A: **PDB Molecule:**laci-type transcriptional regulator;<br>**PDBTitle:** the crystal structure of a laci-type transcription regulator from2 bifidobacterium animalis subsp. lactis dsm 10140
100	d1dbqa_	Alignment	not modelled	99.9	12	**Fold:**Periplasmic binding protein-like I<br>**Superfamily:**Periplasmic binding protein-like I<br>**Family:**L-arabinose binding protein-like
101	c3o74A_	Alignment	not modelled	99.9	11	**PDB header:**transcription<br>**Chain:** A: **PDB Molecule:**fructose transport system repressor frur;<br>**PDBTitle:** crystal structure of cra transcriptional dual regulator from2 pseudomonas putida
102	c3clkB_	Alignment	not modelled	99.9	12	**PDB header:**transcription regulator<br>**Chain:** B: **PDB Molecule:**transcription regulator;<br>**PDBTitle:** crystal structure of a transcription regulator from lactobacillus2 plantarum
103	c2o20H_	Alignment	not modelled	99.9	11	**PDB header:**transcription<br>**Chain:** H: **PDB Molecule:**catabolite control protein a;<br>**PDBTitle:** crystal structure of transcription regulator ccpa of lactococcus2 lactis
104	c4pevB_	Alignment	not modelled	99.9	13	**PDB header:**solute-binding protein<br>**Chain:** B: **PDB Molecule:**membrane lipoprotein family protein;<br>**PDBTitle:** crystal structure of abc transporter system solute-binding proteins2 from aeropyrum pernix k1
105	c2qh8A_	Alignment	not modelled	99.9	11	**PDB header:**structural genomics, unknown function<br>**Chain:** A: **PDB Molecule:**uncharacterized protein;<br>**PDBTitle:** crystal structure of conserved domain protein from vibrio2 cholerae o1 biovar eltor str. n16961
106	c3s99A_	Alignment	not modelled	99.9	9	**PDB header:**lipid binding protein<br>**Chain:** A: **PDB Molecule:**basic membrane lipoprotein;<br>**PDBTitle:** crystal structure of a basic membrane lipoprotein from brucella2 melitensis, iodide soak
107	c3lftA_	Alignment	not modelled	99.9	13	**PDB header:**structure genomics, unknown function<br>**Chain:** A: **PDB Molecule:**uncharacterized protein;<br>**PDBTitle:** the crystal structure of the abc domain in complex with l-trp from2 streptococcus pneumonia to 1.35a
108	c4kmrB_	Alignment	not modelled	99.9	11	**PDB header:**transcription regulator<br>**Chain:** B: **PDB Molecule:**transcriptional regulator, laci family;<br>**PDBTitle:** structure of a putative transcriptional regulator of laci family from2 sanguibacter keddieii dsm 10542.
109	c1zvvA_	Alignment	not modelled	99.9	11	**PDB header:**transcription/dna<br>**Chain:** A: **PDB Molecule:**glucose-resistance amylase regulator;<br>**PDBTitle:** crystal structure of a ccpa-crh-dna complex
110	c4fe4C_	Alignment	not modelled	99.9	10	**PDB header:**transcription<br>**Chain:** C: **PDB Molecule:**xylose operon regulatory protein;<br>**PDBTitle:** crystal structure of apo e. coli xylr
111	c3tb6B_	Alignment	not modelled	99.9	13	**PDB header:**dna binding protein<br>**Chain:** B: **PDB Molecule:**arabinose metabolism transcriptional repressor;<br>**PDBTitle:** structure of the effector-binding domain of arabinose repressor arar2 from bacillus subtilis
112	c2h0aA_	Alignment	not modelled	99.9	13	**PDB header:**transcription<br>**Chain:** A: **PDB Molecule:**transcriptional regulator;<br>**PDBTitle:** crystal structure of probable transcription regulator from2 thermus thermophilus
113	c4rk0C_	Alignment	not modelled	99.9	13	**PDB header:**transcription regulator<br>**Chain:** C: **PDB Molecule:**laci family sugar-binding transcriptional regulator;<br>**PDBTitle:** crystal structure of laci family transcriptional regulator from2 enterococcus faecalis v583, target efi-512923, with bound ribose
114	c4p98A_	Alignment	not modelled	99.9	12	**PDB header:**transport protein<br>**Chain:** A: **PDB Molecule:**extracellular solute-binding protein;<br>**PDBTitle:** abc transporter system solute-bindng protein from conexibacter woesei2 dsm 14684
115	c3jvdA_	Alignment	not modelled	99.9	15	**PDB header:**transcription regulator<br>**Chain:** A: **PDB Molecule:**transcriptional regulators;<br>**PDBTitle:** crystal structure of putative transcription regulation repressor (laci2 family) from corynebacterium glutamicum
116	d1byka_	Alignment	not modelled	99.9	11	**Fold:**Periplasmic binding protein-like I<br>**Superfamily:**Periplasmic binding protein-like I<br>**Family:**L-arabinose binding protein-like
117	c3bilA_	Alignment	not modelled	99.9	14	**PDB header:**structural genomics, unknown function<br>**Chain:** A: **PDB Molecule:**probable laci-family transcriptional regulator;<br>**PDBTitle:** crystal structure of a probable laci family transcriptional regulator2 from corynebacterium glutamicum
118	c3gybB_	Alignment	not modelled	99.9	14	**PDB header:**transcription regulator<br>**Chain:** B: **PDB Molecule:**transcriptional regulators (laci-family<br>**PDBTitle:** crystal structure of a laci-family transcriptional2 regulatory protein from corynebacterium glutamicum
119	c3huuC_	Alignment	not modelled	99.9	9	**PDB header:**transcription regulator<br>**Chain:** C: **PDB Molecule:**transcription regulator like protein;

119	[illegible]	Alignment	not modelled	99.9	9	**PDBTitle:** crystal structure of transcription regulator like protein from2 staphylococcus haemolyticus
120	c3kjxD_	Alignment	not modelled	99.9	15	**PDB header:**transcription regulator **Chain:** D: **PDB Molecule:**transcriptional regulator, laci family; **PDBTitle:** crystal structure of a transcriptional regulator, lacl2 family protein from silicibacter pomeroyi
